# Ethnopharmacological Approaches for Therapy of Jaundice: Part II. Highly Used Plant Species from Acanthaceae, Euphorbiaceae, Asteraceae, Combretaceae, and Fabaceae Families

**DOI:** 10.3389/fphar.2017.00519

**Published:** 2017-08-10

**Authors:** Devesh Tewari, Andrei Mocan, Emil D. Parvanov, Archana N. Sah, Seyed M. Nabavi, Lukasz Huminiecki, Zheng Feei Ma, Yeong Yeh Lee, Jarosław O. Horbańczuk, Atanas G. Atanasov

**Affiliations:** ^1^Department of Pharmaceutical Sciences, Faculty of Technology, Kumaun University Nainital, India; ^2^Department of Pharmaceutical Botany, “Iuliu Haţieganu” University of Medicine and Pharmacy Cluj-Napoca, Romania; ^3^ICHAT and Institute for Life Sciences, University of Agricultural Sciences and Veterinary Medicine Cluj-Napoca, Romania; ^4^Division BIOCEV, Institute of Molecular Genetics, Academy of Sciences of the Czech Republic Prague, Czechia; ^5^Applied Biotechnology Research Center, Baqiyatallah University of Medical Sciences Tehran, Iran; ^6^Institute of Genetics and Animal Breeding of the Polish Academy of Sciences Jastrzebiec, Poland; ^7^School of Medical Sciences, Universiti Sains Malaysia Kota Bharu, Malaysia; ^8^Department of Public Health, Xi’an Jiaotong-Liverpool University Suzhou, China; ^9^Department of Pharmacognosy, University of Vienna Vienna, Austria; ^10^Department of Vascular Biology and Thrombosis Research, Centre for Physiology and Pharmacology, Medical University of Vienna Vienna, Austria

**Keywords:** jaundice, bilirubin, oxidative stress, traditional use, phytoconstituents, serum enzymes, alkaline phosphatase

## Abstract

In many developing countries, jaundice is the common symptom of hepatic diseases which are a major cause of mortality. The use of natural product-based therapies is very popular for such hepatic disorders. A great number of medicinal plants have been utilized for this purpose and some facilitated the discovery of active compounds which helped the development of new synthetic drugs against jaundice. However, more epidemiological studies and clinical trials are required for the practical implementation of the plant pharmacotherapy of jaundice. The focus of this second part of our review is on several of the most prominent plants used against jaundice identified in the analysis performed in the first part of the review viz. *Andrographis paniculata* (Burm.f.) Nees, *Silybum marianum* (L.) Gaertn., *Terminalia chebula* Retz., *Glycyrrhiza glabra* L. and some species of genus *Phyllanthus*. Furthermore, we discuss their physiological effects, biologically active ingredients, and the potential mechanisms of action. Some of the most important active ingredients were silybin (also recommended by German commission), phyllanthin and andrographolide, whose action leads to bilirubin reduction and normalization of the levels of relevant serum enzymes indicative for the pathophysiological status of the liver.

## The Medicinal Plants of Prime Importance for the Treatment of Jaundice

**FIGURE 1:**
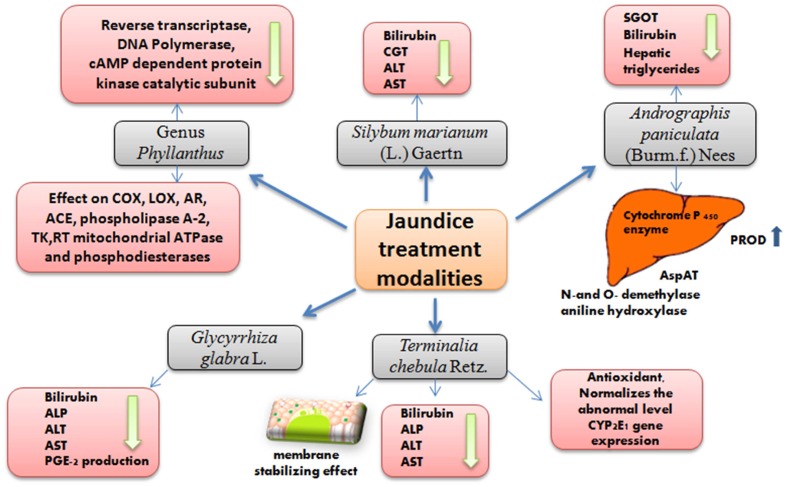
Overview of observed effects associated with the use of the reviewed plants for jaundice treatment.

In the first part of the review, we presented an overview of the history, symptoms and causes of jaundice and the significance and diversity of medicinal plants used in its treatment. We also presented an exhaustive list of 207 plant species from 20 countries used for the treatment of jaundice. These plants were mainly indicated by different ethnopharmacological or ethnobotanical surveys. Based on their most popular use, several of the 207 plants classified in five different families are described in the current review based on their therapeutic profiles with special reference to jaundice and hepato-protective mechanism (**Figure 1**).

### *Andrographis paniculata* (Burm.f.) Nees

**FIGURE 2:**
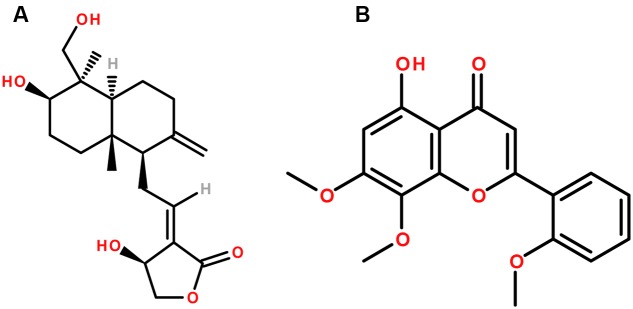
The chemical structures of phytoconstituents of *Andrographis paniculata* **(A)** Andrographolide and **(B)** Andrographin.

*Andrographis paniculata* (Burm.f.) Nees is a medicinal plant which belongs to the family Acanthaceae. The major biologically active chemical constituents of the plant are diterpene lactones, either free, or glycosidated. These include andrographolide, deoxyandrographolide, neoandrographolide, andrographiside, andrographanoside, etc. (**Figure 2**) (Xu, 1986; Thai Pharmacopoeia Committee, 1995; WHO, 1999). It is used as a cure for a broad spectrum of diseases and utilized traditionally for centuries in folk medicine, mainly in Asia (Jarukamjorn et al., 2006). Aerial parts of *A. paniculata* are included in traditional remedies, and are being used for a broad range of disorders, more specifically as hepatic stimulant and hepatoprotective agent along with other liver disorders and jaundice (Kapil et al., 1993; Trivedi and Rawal, 2000). The medicinal effect of the aerial parts of the plant is represented by their *in vitro* and *in vivo* anti-hepatotoxic activities (Gupta et al., 1990; Chander et al., 1995). These studies demonstrated marked effect of the *A. paniculata* and its diterpenes andrographolide and neoandrographolide on the alkaline phosphatase (ALP), serum lipoprotein-X, GPT, GOT, bilirubin and also the effect of *A. paniculata* on the enterotoxin in animal models. These studies also showed promising protective effects of the diterpenes andrographolide and neoandrographolide in hepatic damage. However, further clinical studies with sufficient number of subjects are required to further prove the efficacy of these compounds in humans.

Various studies have been carried out to explore the therapeutic effect of this plant, particularly its antiviral (Calabrese et al., 2000), anti-inflammatory (Shen et al., 2002), immune stimulatory (Puri et al., 1993; Iruretagoyena et al., 2004), and anti-cancer effects (Kumar et al., 2004). *A. paniculata* extract, and in particular its active compound andrographolide and its related analogs were shown to exhibit effects on various hepatic enzymes like *N*-and *O*-demethylase, aniline hydroxylase (Choudhury and Poddar, 1984), AspAT, alanine aminotransferase (ALT) (Trivedi and Rawal, 2000), DT-diaphorase (DTD) and glutathione *S*-transferase (GST) (Singh et al., 2001). The aqueous extract of *A. paniculata* significantly increased the pentoxyresorufin *O*-dealkylase (PROD) activity, suggesting that *A. paniculata* constituents might affect hepatic cytochrome P-450 enzyme (Jarukamjorn et al., 2006).

CCl_4_ induced hepatotoxicity in mice was reduced by methanol extract of *A. paniculata* and the histopathological liver changes were reversed (Handa and Sharma, 1990), as well as the elevated concentrations of different enzymes like SGOT; bilirubin and hepatic triglycerides were reduced by andrographolide (Handa and Sharma, 1990; Sharma et al., 1991). Andrographolide also exerted a prominent protective effect against hepatotoxicity caused by various substances viz. D-Gal N (Saraswat et al., 1995), ethanol (Pramyothin et al., 1994), paracetamol (Visen et al., 1993), and CCl_4_ (Kapil et al., 1993). Comparison showed that andrographolide exhibits higher efficiency than the standard hepatoprotective agent silymarin (Kapil et al., 1993; Visen et al., 1993). However, it is still not clear if the extract, or the pure substance has higher efficiency based on their inhibition on CCl_4_ and paracetamol toxicity (Choudhury and Poddar, 1984; Visen et al., 1993). This can be due to presence of several complex phytochemicals in the extract, which can lead to synergistic, or antagonistic effects during bioactivation and detoxification (Singh et al., 2001).

A report on modulation potential of phases I and II enzymes and antioxidant enzymes by *A. paniculata* revealed a remarkable effect on hepatic metabolic enzymes. The activities of DTD and GST were increased in mouse liver and other organs by an 80% hydroalcoholic extract of the plant and thus strengthening the xenobiotic metabolism toward detoxification, which plays an important role in chemoprevention and cytoprotection (Singh et al., 2001). The effect of the plant was also correlated with numerous GST, DTD inducing phytoconstituents, having an important role against chemical-induced carcinogenesis (Singh et al., 2001). The intracellular GSH concentration, which is of major importance for detoxification of xenobiotics is also modulated by *Andrographis* usage (Ketterer, 1988; Meister, 1994).

In a clinical study by Chturvedi et al. (1983) outstanding results by using *A. paniculata* for the treatment of infectious hepatitis were achieved. Improvements in appetite, jaundice, fever and epigastric discomfort were observed in all patients within 4 weeks. A significant reduction in serum bilirubin level (up to 10-fold) was recorded as well. Apart from this, ALP, SGPT and SGOT, were also significantly improved, suggesting that *A. paniculata* might be considered as the cheapest and the most beneficial cure for infectious hepatitis (Chturvedi et al., 1983). Although these results appear promising, it should be considered that this study conducted more than three decades ago had low number of studied subjects. The lack of statistical power and the insufficient data by the former experiments require the conduction of larger clinical trials to elucidate the effects of the active compounds. It should be considered that next to the major constituents andrographolide and andrographin there may be also other compounds modulating the bioactivities of the plant. Therefore, further studies on the synergistic and antagonistic effects of the plant compounds combined with knowledge about their deeper molecular mechanisms are required to unravel the mode of action of *A. paniculata*.

In brief, *A. paniculata* seems effective in various liver diseases such as viral and toxic hepatitis and intra and extra hepatic cholestasis, that would reduce jaundice (Deng et al., 1982; Handa and Sharma, 1990; Visen et al., 1993). However, there are also reports about its compounds cytotoxicity (Nanduri et al., 2004) and toxicity for the male reproductive system (Akbarsha and Murugaian, 2000). Therefore, further clinical studies are required to establish the safety and clinical efficacy of the long term use.

## Genus *Phyllanthus*

The genus *Phyllanthus* belongs to the Euphorbiaceae family and is widely distributed throughout the tropical and subtropical zones being described for the first time in 1773 by Linnaeus. The genus comprises of about 550–1200 species (Unander et al., 1990, 1991, 1992, 1995; Mabberley, 2008; Cruz-Vega et al., 2009). A number of species of this genus have been used since ancient times to treat a broad spectrum of diseases such as the hepatitis B virus (HBV) infection, bone disorders (Piva et al., 2009), diabetes (Kusirisin et al., 2009), intestinal infections and disturbance of the kidney and urinary bladder (Morton, 1981; Oliver-Bever, 1983; Unander et al., 1990, 1991, 1992, 1995; Calixto et al., 1998; Cruz-Vega et al., 2009). Evidences for utilization of this genus are present in the aboriginal texts of Ayurveda (also known as “*Ancient Science of Life*”), which is one of the ancient traditional medicine systems of the world, native to India for more than 2000 years. Apart from India, these species are also used in other locations viz. Central and South America, Philippines, Guam, Nigeria, Cuba, China, and Africa (Thyagarajan et al., 1988).

**FIGURE 3:**
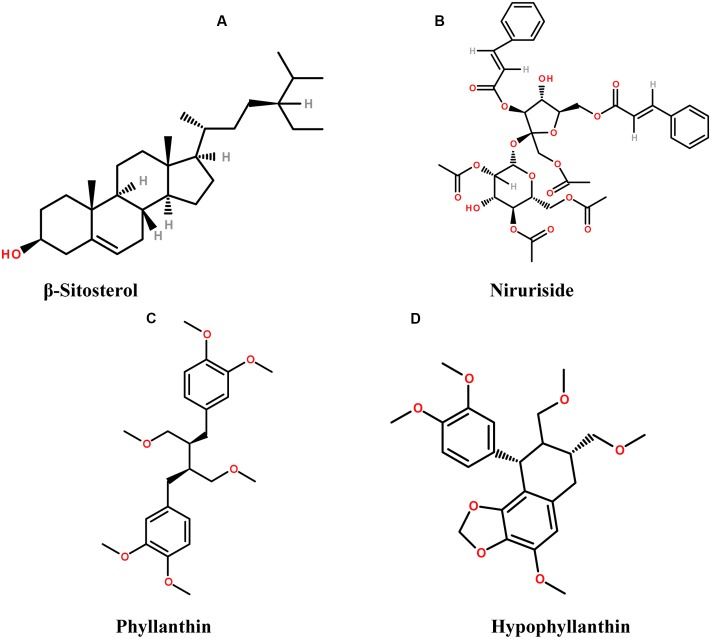
The chemical structures of phytoconstituents present in species from *Phyllanthus* genus **(A)** β-sitosterol, **(B)** Niruriside, **(C)** Phyllanthin, and **(D)** Hypophyllanthin.

A variety of bioactive molecules have been isolated and characterized from a large number of species of this genus (**Figure 3**). Phytochemical studies revealed the presence of lignans, alkaloids, tannins, lactones, steroids, and flavonoids (Pettit et al., 1982a,b; Foo and Wong, 1992; Bachmann et al., 1993; Foo, 1995; Filho et al., 1996; Calixto et al., 1998). Therapeutic activities have been reported from phytosterols like β-sitosterol, stigmasterols, and campesterol, present in *Phyllanthus* species having antinociceptive action among other effects. Furthermore, rutin in *Phyllanthus emblica, P. niruri*, and *P. amarus* is associated with analgesic and anti-inflammatory activity (Alcaraz and Jiménez, 1988; Pathak et al., 1991; Santos et al., 1994); ellagic acid found in *P. emblica* and *P. niruri* acts as an aldose reductase inhibitor (Shimizu et al., 1989; Unander et al., 1991); geraniin has antiallergic, analgesic and ACE-inhibitor activity; and quercetin has mitochondrial ATPase, phosphorylase and tyrosine kinase inhibition, analgesic activity, cyclooxygenase (COX) inhibition; phospholipase A-2 inhibition and mutagenic effect on bacteria (Suolinna et al., 1974; Beretz et al., 1978; Shisheva and Shechter, 1992; Duarte et al., 1993; Lindahl and Tagesson, 1993; Morales and Lozoya, 1994; Filho et al., 1996; Qian-Cutrone et al., 1996; Calixto et al., 1998; Ullah et al., 2013). Niruriside present in *P. niruri* has HIV transcriptase inhibitory activity (Hussain et al., 1995; Qian-Cutrone et al., 1996). Positive effect in the treatment of genitourinary infections, HBV infection, diabetes and airborne disease management are attributed as well to *P. niruri* L. (Perry and Metzger, 1980; Oliver-Bever, 1983; Calixto et al., 1998). *In vitro* studies showed that the reverse transcriptase from human immuno deficiency virus type-I (HIV-RT) was inhibited by the aqueous extract of *P. niruri* (Ogata et al., 1992; Naik and Juvekar, 2003).

*Phyllanthus niruri*, is an erect, small herb, indigenous to Amazon rainforest, South India, China and South Africa (Girach et al., 1994). *P*. *niruri* (wh. pl.) is used in different disease conditions like diabetes, jaundice, tumors, kidney stones, influenza etc. Moreover, the use of this plant for prevention of hepatotoxicity, viral, bacterial infection and inhibition of HBV is also reported (Chopra et al., 1956; Bagalkotkar et al., 2006). *P. niruri* is present worldwide in different traditional systems of medicine. It is known by several names in different medicinal systems such as “Tamalaki” or “*Bhumyan amalaki*” in Ayurveda, which means “resembling Indian gooseberry” (Indian gooseberry is *P. emblica* which is known as *‘Amalaki’* in Sanskrit), *P. niruri* also known as “*Dukong*” in Malaysia, “*Chanca Piedra*” in Spanish, which means ‘breaker of stone’ due to its lithotriptic activity that has been used as an effective remedy to eliminate different types of stones like, kidney and gall stones (Bagalkotkar et al., 2006). It has also exhibited non-concentration dependent inhibition on CaOX crystals formation (Campos and Schor, 1999). The plant is also known as “*Quebra Pedra*” in Brazil, where it is considered as an excellent remedy for renal disorders, bladder associated infections and hydropsy (Dias et al., 1995; Wang et al., 1995; Wang, 2000). It is also known as “*Pitiriasi*,” or “*Budhatri*” and is used as a household remedy for anemia, jaundice, tuberculosis, extreme thirst, respiratory disorders etc. in India (Dhar et al., 1968). The work by Otawa in 1891 led to the isolation of the compound phyllanthin (Row et al., 1966). Phyllanthin and hypophyllanthin are lignans and the hepatoprotective and anti-genotoxic activity of *P. niruri* is attributed to their presence (Row et al., 1966). However, the hepatoprotective activity is not exclusively due to the presence of hypophyllanthin and phyllanthin as these constituents are present only in *P. amarus* (Khatoon et al., 2006) but other *Phyllanthus* species such as *P. polyphyllus*, *P. acidus*, and *P. fraternus* also exhibit the hepatoprotective activity and the presence of hydroxyl rich compositions and antioxidant activity may be correlated with the hepatoprotective effect (Mao et al., 2016).

The first medically oriented research on *Phyllanthus* plants was conducted by Indian and Brazilian teams due to the traditional use of the plants by indigenous people in these areas (Unander and Blumberg, 1991; Bagalkotkar et al., 2006). The effect of *P. niruri* on jaundice among children was reported by a group of Indian scientists (Dixit et al., 1982; Bagalkotkar et al., 2006). Damage of hepatic mouse tissues was found to be counteracted by the protein fraction of *P. niruri*. Some of the potent antioxidants from this plant such as rutin, quercetin are also beneficial in colonic inflammation treatment (Gálvez et al., 1995, 1997; Cruz et al., 1998; Sanchez de Medina et al., 2002); gallocatechin, isolated by tissue culture of *P. niruri* (Ishimaru et al., 1992) and catechins in general have suppressive effect on the growth of hepatic and colon epithelial cancer cell lines (Uesato et al., 2001). Moreover, the flavone glycoside nirurin [5,6,7,4-tetrahydroxy-8-(3-methylbat-2-enyl)] and some volatile constituents were also reported as present in the plant (Bagalkotkar et al., 2006). *P. niruri* gained particular attention globally due to its anti HBV activity during late 1980s (Venkateswaran et al., 1987).

The extract of *P. amarus* Schumach. & Thonn. demonstrated steady inhibition on the HBV surface antigen *in vitro*. Most of the plant extracts of this genus are capable of acting as reverse transcriptase inhibitors and DNA polymerase inhibitors and, thus, preventing the replication of HBV (Thyagarajan et al., 1988; Unander and Blumberg, 1991; Ogata et al., 1992; Unander et al., 1995; Lee et al., 1996; Calixto et al., 1998; Padmalatha et al., 2009). The chemical constituents responsible for HBV suppression are still not well known, although ellagic acid may have a role for this effect. Further reports showed that hydrolysable fraction from *P. amarus* acts via inhibition of cAMP dependent protein kinase catalytic subunit (Polya et al., 1995). Clinical studies revealed that *P. amarus* extract had very little, or no adverse effect on the patients (Thyagarajan et al., 1988; Wang et al., 2005).

The toxic and therapeutic effects of other plants of this genus like *P. urinaria* L. and *P. niruri* were examined in 123 patients and significant improvements were observed in those with chronic hepatitis (Wang et al., 2005). In a study performed by Yeh et al. (1993), a reversible inhibition of cellular proliferation and suppression of HBV surface antigen was observed in human hepatoma cell lines (HepA2). The biological effect of *P. amarus* extract and its medical use in jaundice treatment was explained by reducing the mRNA level of the HBV surface antigen (Wang et al., 2005). The HBV suppression by plant extracts of *Phyllanthus* origin is a well-known fact. However, the discovery of active molecules and their mechanism of action is a matter of future studies. This will allow improving liver function and resolving jaundice by their more efficient analogs, or by combination with other compounds. In addition to the spectrum of favorable activities of this genus, the clastogenic effect of nickel chloride on mouse bone marrow cells is antagonized by *P. emblica* and *P. niruri* extracts (Agarwal et al., 1992); and potential hypoglycemic, diuretic and hypotensive effects of *P. amarus* were also observed in a human clinical study (Srividya and Periwal, 1995). The hepatoprotective activity of different *Phyllanthus* species was also evaluated in animal models (Syamasundar et al., 1985; Dhir et al., 1990; Gulati et al., 1995; Prakash et al., 1995). Liver injury induced by CCl_4_ was counteracted by *P. niruri* and *P. urinaria*, but not by *P. simplex*, and normalization of the elevated serum levels of transaminases (SGOT and GPT) in rat liver was observed (Syamasundar et al., 1985; Prakash et al., 1995); quercetin, which is one of the main component of *P. emblica*, exhibited hepatoprotective effect after paracetamol and country-made liquor intake in mice, and *P. niruri* also led to similar result (Umarani et al., 1985; Gulati et al., 1995; Unander et al., 1995). It is assumed that most of the phytoconstituents present in this genus, as described earlier, interact with key regulatory enzymes viz. COX, LOX, AR, ACE, phospholipase A-2, tyrosine kinase (TK), reverse transcriptase (RT) mitochondrial ATPase and phosphodiesterases (Calixto et al., 1998). The clinical study of Thyagarajan et al. (1988) found weaker responses to HBV treatment by *P. amarus* extracts in males vs. females, however, the number of individuals was insufficient for statistical significance of this difference.

In conclusion, it is evident that the plants of genus *Phyllanthus* have a great potential as therapeutics against jaundice. However, there is a need of proper clinical trials aimed toward the establishment of safety and efficacy of the genus *Phyllanthus* species in a standardized way.

## *Silybum marianum* (L.) Gaertn. (milk thistle)

**FIGURE 4:**
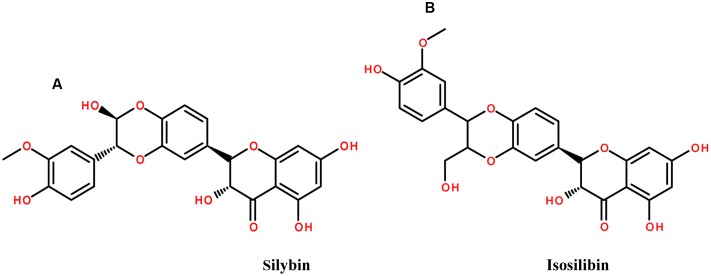
The chemical structures of phytoconstituents of Milk thistle **(A)** Silybin and **(B)** isosilibin.

*Silybum marianum* (L.) Gaertn. (*Carduus marianus* L.), belonging to the family Asteraceae, or Compositae, has been used for more than 2000 years, in particular as a remedy for hepatobiliary disorders since 16th century (Flora et al., 1998; Schuppan et al., 1999). The plant is commonly known as the milk thistle, Our lady’s thistle and St. Mary’s thistle (Schuppan et al., 1999; Wellington and Jarvis, 2001). One of the most important hepatoprotective agent, silymarin is obtained from milk thistle fruits and seeds. The plant extract has a variety of phytoconstituents including silybin, or sometimes incorrectly called silibinin, which are flavonolignans, as well as isosilibin, silychristin, and silidianin (**Figure 4**). The plant is widely spread in the United States and it became officially used in clinical practice after 1969 (Morazzoni and Bombardelli, 1995; Flora et al., 1998; Šimanek et al., 2000).

A study by Flora et al. (1996) revealed that 31% of patients were taking OTC milk thistle as an alternative agent for liver diseases. Dioscorides, the famous Greek herbalist, wrote about the use of tea from milk thistle seed against snake poison/bite (Greive, 1981). The use of milk thistle against hyperbilirubinemia was described by Pliny The Elder (AD 23–79) (Foster, 1991). Later, in 1596, Gerarde considered milk thistle as the best remedy against black bile or melancholy (Hobbs, 1987). Furthermore, in 1787 the seeds and the roots of the plant were noted as an excellent remedy to treat liver and spleen obstruction and to cure jaundice along with expelling stones (Greive, 1981).

General features of flavonoids are their membrane stabilizing effect and free radical capture, which defines their biological activity. Silybin, being a flavolignan is considered as the most efficient phytochemical responsible for different therapeutic activities of the plant viz. hepatoprotective, antiangiogenic, chemoprotective etc. (Gazak et al., 2007). Fruits and seeds are considered to be the richest in active constituents along with leaves (Harnisch and Stolze, 1983; Hobbs, 1987; Flora et al., 1998). Silybin is the major component of silymarin, constituting about 50–70% of the silymarin extract (Loguercio and Festi, 2011). The effect of silymarin against liver diseases of different etiology such as hepatobiliary diseases was reported during 1960s (Wagner et al., 1974; Luper, 1998; Pradhan and Girish, 2006). Silymarin has been extensively studied for its different pharmacokinetic and pharmacodynamic properties, and mechanism of liver protective action (Ramellini and Meldolesi, 1974; Miadonna et al., 1987; Feher et al., 1989; Barzaghi et al., 1990; Wellington and Jarvis, 2001).

Moreover, the effect of silymarin on liver cirrhosis was investigated in several randomized double blind clinical studies (Salmi and Sarna, 1982; Feher et al., 1989; Ferenci et al., 1989; Bunout et al., 1992; Parés et al., 1998). Some studies revealed a significant increase in the patient survival rate after the treatment with silymarin (Ferenci et al., 1989; Parés et al., 1998). It was also reported that slightly elevated levels of serum bilirubin are normalized by silymarin treatment and CGT, ALT, and AST are decreased significantly (Feher et al., 1989). However, another study showed that 420 mg/day dose of silymarin did not inflict significant effect on the serum level of total bilirubin, ALP and, AST, in patients with primary biliary cirrhosis (Angulo et al., 2000). The opposite result was obtained at the same dose, i.e., 420 mg/day, in a randomized, multicentric, double blind study with larger number of patients (*n* = 59). Significant reduction of serum AST and bilirubin was recorded as comparable to placebo in patients with acute viral hepatitis A or B (Magliulo et al., 1978). Serum ALT level was decreased significantly in patients with chronic hepatitis by studies including 180 patients with chronic persistent hepatitis (Tănăsescu et al., 1987).

The daily dose of 12–15 g of crude herb, or 200–400 mg of silymarin (counted as silybin) is recommended by German commission E (Meyer et al., 1999). Tablets, or capsules with a dose of 70 or 140 mg, or milk thistle fruit infusion are also used (Fleming, 1998; Meyer et al., 1999; Wellington and Jarvis, 2001). The drug should be avoided for children below age of 12 years due to lack of proper clinical tests for its effect. Several other parameters like enhancement of SOD activity, as well as SOD expression in erythrocytes and lymphocytes along with the increase of glutathione and glutathione peroxidase levels are also associated with silymarin intake (Wellington and Jarvis, 2001). Consequently, silymarin is becoming a more popular OTC herbal preparation in Europe due to its liver regeneration properties. A large number of patients with different liver disease are taking this drug with other prescribed medications in United States as well (Leng-Peschlow, 1994; Morazzoni and Bombardelli, 1995; Schuppan et al., 1999; Šimanek et al., 2000; Wellington and Jarvis, 2001).

## *Terminalia chebula* Retz.

**FIGURE 5:**
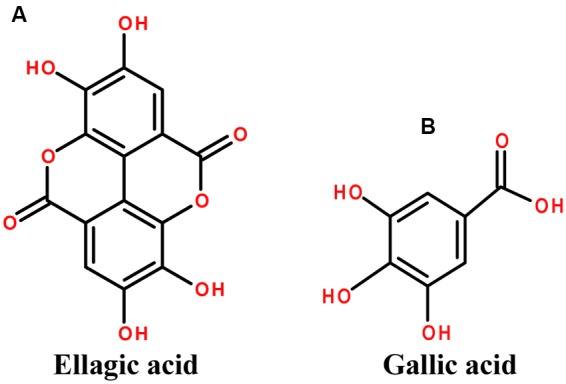
The chemical structures of phytoconstituents of *Terminalia chebula* **(A)** Ellagic acid and **(B)** Gallic acid.

*Terminalia chebula* Retz. originates from India (Choi et al., 2015) and belongs to the family Combretaceae commonly known as “chebulic myrobalan” and “*Haritaki*” (Tasduq et al., 2006; Yeasmin et al., 2016). *T. chebula* contains significant amount of phenolic and flavonoid compounds. Some of the main constituents are 2,4-chebulyl-β-D-glucopyranose, ellagic acid, gallic acid and chebulic ellagitannins (**Figure 5**) (Juang et al., 2004; Panunto et al., 2010; Li et al., 2014).

The plant is well recognized for its various medicinal uses, and it is one of the main ingredient of the important Ayurvedic formulation “*Triphala*” (three myrobalan fruits) (Anand et al., 1994). Ayurvedic Pharmacopoeia of India prescribes this formulation to cure kidney and liver dysfunctions (Chatterjee and Pakrashi, 1991; Mhaskar et al., 2000). The use of dried ripe fruit of *T. chebula* is reported in Ayurveda as antitussive, cardiotonic, homeostatic, diuretic and laxative (Lee H.-S. et al., 2005; Sarkar et al., 2012). There are several formulations with *T. chebula* as the main ingredient, for example the official formulation in Ayurvedic Pharmacopoeia of India named “*Triphala Ghrita*”, which is a mixture of *T. chebula*, *T. bellirica*, *Phyllanthus emblica* and 19 other compounds found to have beneficial effect on jaundice (*Kamala*). The plant is also the main ingredient of the polyherbal hepatoprotective drug HP-1 (Tasaduq et al., 2003).

Together with the suppressive effect on hepatic fibrosis, the fruit extract of *T. chebula* inhibits the lipid peroxidase and affects the iron chelation associated protein oxidation. Reduction in ALT, AST, ALP and total bilirubin level indicates protection of liver damage (Sharma and Rathore, 2010; Sarkar et al., 2012; Yeasmin et al., 2016). The antioxidative role of the 70 % methanolic extract is due to its chelating properties for iron ion and thus decreasing the toxicity in iron overload states (Harrison, 1977; Sarkar et al., 2012). Aqueous fruit extract of *T. chebula* was tested against severe acute liver injury by t-BHP (*tert*-butyl hydroperoxide) in mice (Choi et al., 2015). The *t*-BHP causes rigorous necrosis, damage of hepatic tissues and significant elevations of serum enzymes like LDH, AST, and ALT. *T. chebula* fruit extract normalizes the liver enzyme levels and exhibits antioxidant effects overall providing liver protection (Owoyele et al., 2001; Fu et al., 2010; Choi et al., 2015; Yeasmin et al., 2016).

*T. chebula* extract also normalizes the abnormal level of CYP2E_1_ gene expression, which is mainly activated during drug metabolism process (Rush et al., 1985; Choi et al., 2015). Altogether with the hepatoprotective effect in different models (Tasduq et al., 2006; Sarkar et al., 2012), the immune modulatory action mediated by chebulagic acid in *T. chebula* is also reported (Lee S. et al., 2005). Additionally, due to its antioxidant activity and bilirubin level lowering effect *T. chebula* extract ensures hepatoprotection against paracetamol-induced damage. The reduction in serum bilirubin level is the most important evidence supporting the traditional use of the plant against jaundice.

Hepatotoxicity associated with prolonged use of rifampicin (RIF), isoniazid (INH) and pyrazinamide (PZA), which are used in a combination for the chemoprophylaxis and treatment of tuberculosis (Wong et al., 2000), is common. The side effects of anti-tuberculosis drugs on liver were reduced after the use of *T. chebula* fruit extract containing 0.250% chebuloside on an oral administration for 12 weeks in rats (Tasduq et al., 2006). Membranes (cytoplasmic and microsomal) were found as the main target of action for *T. chebula* extract. Hepatoprotective effect of *T. chebula* is also based on effects on the Na^+^, K^+^ -ATPase and CYP2E_1,_ similarly to silymarin (Mourelle et al., 1989; Tasduq et al., 2006).

## *Glycyrrhiza glabra* L.

Genus *Glycyrrhiza* belongs to the family Fabaceae, and consists of around 30 species. The plants of this genus are perennial herbs and are native to Mediterranean region and Asia, from Iran to Southern Russia. They are also cultivated throughout Europe and Asia (Blumenthal et al., 2000; Asl and Hosseinzadeh, 2008). The most common plant of this genus is *Glycyrrhiza glabra* L. also known as licorice. It has been utilized for relief of catarrh of the respiratory organs since ancient Egyptian times and described in the Ebers papyrus (1552 B.C.) and Codex Hammurabi (2100 B.C.). It also appears in “De Materia Medica” of Dioscorides (40–90 A.D.) in Rome and “De Causis Plantarum” and “De Historia Plantarum” of Theophrastus (371–286 B.C.) in Greece. According to Dioscorides manuscript entitled “Glukoriza” (sweet root), the expressed sap of its root is used for the liver, stomach, and kidney related ailments (Shibata, 2000). Flavonoid content from the root was found to exert antiulcerogenic and spasmolytic activity (Kitagawa et al., 1994; Fukai et al., 1996; Evans, 2009). Several isoflavonoid derivatives such as shinpterocarpin, glabrone, glabrene, glabridin, lico-isoflavones A and B etc. are also present in licorice (Williamson, 2003; Asl and Hosseinzadeh, 2008). The ancient medicinal use of licorice has been documented in the Chinese medical book “Shang-Han-Za-Bing-Lun” as well. Among 113 prescriptions of Shang-Han-Lun, 80% contain licorice as a significant constituent (Shibata, 2000). The cultivation of this plant in England has been traced back to the sixteenth century (Evans, 2009). The plant is utilized for its medicinal activity since 500 BC and described as ‘the grandfather of the herbs’ (Ody, 2000). It is known under different names like licorice, gancao, yasthi-madhu, kanzoh and sweet root (Blumenthal et al., 2000; Nomura et al., 2002; Asl and Hosseinzadeh, 2008). *G. glabra* L., *G. bucharica* Regel, *G. foetida* Desf., *G. aspera* Pall., *G. echinata* L., and *G. inflata* Batalin are some of the species of this genus among which three have varieties: *G. glabra* viz. *G. glabra* var *typica* (Spanish and Italian licorice), *G. glabra* var *glandulifera* (Russian licorice) and *G. glabra* var *violacea* (Turkish licorice) (Nomura et al., 2002). Substantial research has been carried out for the main medicinal effects of licorice since 1990 largely by the Japanese scientists, since the drug is widely used in the traditional medicine of Japan, brought earlier from China (Wang et al., 1996; Arase et al., 1997; Shibata, 2000; van Rossum et al., 2001; Hidaka et al., 2007; Evans, 2009; Eerdunbayaer et al., 2014; Ohno et al., 2014).

**FIGURE 6:**
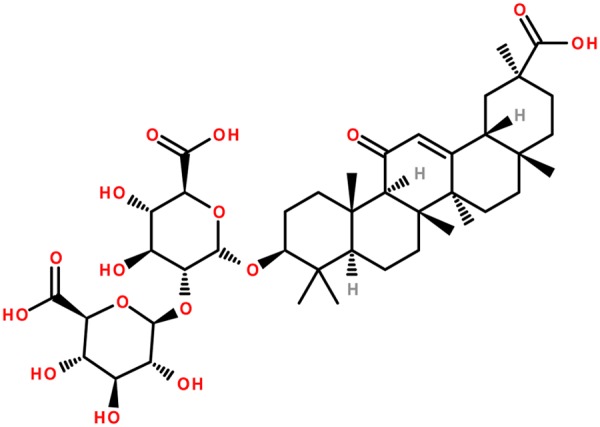
Chemical structure of glycyrrhizin (glycyrrhizic acid): the main active constituent of glycyrrhiza.

The sweet taste of licorice is due to the triterpenoid saponin known as glycyrrhizin (glycyrrhizic acid) (**Figure 6**). Further, the glycyrrhizinic acid is diglucopyranosiduronic acid of the glycyrrhetinic acid, which has a triterpenoid structure (Kokate et al., 2003; Evans, 2009). Flavonoids are the cause of the yellow color of licorice, and they were recognized in 1978 for their inhibitory effect on gastric acid secretion (Dastagir and Rizvi, 2016). Flavonoids include liquiritin, isoliqueritin (a chalcone), liquiritigenin, neoliuirtin, rhamnoliuirtin and others (Williamson, 2003; Asl and Hosseinzadeh, 2008).

The leaves, roots and rhizomes of licorice have been used traditionally for different purposes including wound healing (Dafni et al., 1984), cough (Fujita et al., 1995) cystitis (Yarnell, 1997), tuberculosis (Arseculeratne et al., 1985), gastric ulcer (Varshney et al., 1983) and diabetes (Rajurkar and Pardeshi, 1997). Licorice shows different pharmacological activities such as hepatoprotective (Nakamura et al., 1985; Lin and Tome, 1988; Nose et al., 1994; Lin et al., 1999; Al-Qarawi et al., 2001; Jeong et al., 2002; Lee et al., 2009; Huo et al., 2011), anti-inflammatory (Finney and Somers, 1958; Ohuchi and Tsurufuji, 1982; Yu et al., 2015; Yang et al., 2016), antiviral, antimicrobial (Shebl et al., 2012; Sabouri Ghannad et al., 2014; Wang et al., 2015) and immunomodulatory effect (Kimura et al., 1992; Tandon et al., 2002; Raphael and Kuttan, 2003). Glycyrrhizin is widely used for treatment of different types of hepatitis (Yamamoto et al., 1958; Suzuki et al., 1977). However, there are reports of aldosteronism effect when used in enormous doses (Ulmann et al., 1975; Epstein et al., 1977; Ishikawa and Saito, 1980). Pharmacokinetic studies of glycyrrhizin were also carried out in patients with hepatitis and liver cirrhosis and a monophasic decline in plasma concentration of glycyrrhizin was found with 6.0 h elimination half-life (*t*_1/2_) and 7.9 ml h^-1^ kg^-1^ total body clearance (CLtot) (Tanaka et al., 1993; Yamamura et al., 1995). Another pharmacokinetic study in rats with D-Gal N induced hepatic disease didn’t show significant change in bioavailability of glycyrrhizin (Wang et al., 1996).

Use of licorice extract for peptic ulcer was reported by Revers in 1946. Studies have been conducted extensively to investigate the effect of glycyrrhizin alone, or in combination with other drugs against chronic hepatitis (Takahara et al., 1994; Arase et al., 1997; Lau et al., 2000; Tandon et al., 2001), and subacute hepatitis (caused mainly by HBV and HEV). Duration of illness and other fatal complications of subacute liver failure due to HEV are also reduced by intravenous glycyrrhizin therapy along with marked decline in elevated bilirubin concentration (Tandon et al., 2002). One of the popular preparations containing glycyrrhizin, L-cysteine and glycine that have been used in Japan for more than six decades is with the trade name of “Stronger Neo-Minophagen C” (SNMC). Initially the preparation was used as an antiallergic and antidote to toxic substances. Later on, the medication was used intravenously for chronic hepatitis, plummeting serum AST (GOT) and ALT (GPT) levels in patients. A double-blind clinical trial of SNMC for treatment of chronic hepatitis was carried out by Suzuki and coworkers and observed significant decline in plasma transaminase activity and improvement of the liver cells in histological samples from the SNMC treated group (Suzuki et al., 1977). Intravenously administered glycyrrhizin is rapidly eliminated from sera, and is transformed to glycyrrhetinic monoglucuronide by means of hepatic β-glucuronidase. The intravenous administration of SNMC also led to significant decline of elevated AST and ALT levels in hepatitis patients. Significant suppression of the release of AST from hepatocytes was also found in isolated rat hepatocytes by glycyrrhizin and glycyrrhetinic acid incubated with anti-liver cell membrane antibody (Shiki et al., 1992; Shibata, 2000). Yoshikawa et al. (1997) using an antigen-specific murine CD4+ T hybridoma cell line, showed that glycyrrhizin restrains immune-mediated cytotoxicity against hepatocytes, and thus explaining the reduction of AST and ALT elevated plasma levels. These were the results of apoptosis of hepatocytes resulting from liver injury (Hiramatsu et al., 1994; Mita et al., 1994). Anti-Fas antibody-induced elevation of ALT in mice was counteracted by glycyrrhizin and it was hypothesized that ALT decrease in the chronic hepatitis C virus (HCV) patients treated with SNMC might be due to the inhibition of Fas-mediated hepatic injury. The anti-inflammatory effect of SNMC is believed to be mediated by its membrane protecting activity and it is associated with a lower transaminase level in chronic hepatitis patients (Watari, 1973; Utsumi, 1984). Glycyrrhizic acid inhibits cisplatin efflux from the cells and reverses cisplatin resistance in HCC cell (Wakamatsu et al., 2007). The glycyrrhizic acid effect is associated with reduced immunosuppression, cell cycle arrest, induction of autophagy and apoptosis of the HCC cells (Satomi et al., 2005). The mechanism of action of glycyrrhizic acid is via its binding to glycyrrhizic acid membrane receptors on the hepatocytes and glycyrrhetinic acid-modified novel drug delivery system for HCC was consequently developed based on the promising activity pattern of this molecule (Cai et al., 2016). Several clinical trials were also performed on SNMC to see the effect of glycyrrhizin in hepatitis and hepatocarcinogenesis (Suzuki, 1983; Hino et al., 1994; Arase et al., 1997; van Rossum et al., 1999; Ikeda et al., 2006; Veldt et al., 2006). These trials revealed the effectiveness of SNMC in the prevention of liver carcinogenesis and the effect of glycyrrhizin in decreasing the HCC, improvement of plasma transaminase activity and effect on chronic HCV infected patients with non response toward interferon. Further, glycyrrhizin therapy exhibited normalization of ALAT levels and showed less incidence of HCC.

Licorice also possesses preventive role on the development of hepatocellular carcinoma (HCC) in HCV-associated chronic hepatitis patients (Arase et al., 1997; Miyakawa and Iino, 2001). Patients with long-term abnormal serum levels of α-fetoprotein (AFP) after transfusion have high probability of HCC and histological aggravation (Ikeda et al., 1993). They could benefit using licorice to maintain normal liver function.

Based on their biological activity and efficiency in the cure of some hepatic diseases, including HCC, there is increasing interest toward the use of herbs and natural products, in the treatment and prevention of these disorders. The reviewed plants are widely used in hepatic disorders treatment and their biologically active components have been extensively studied. However, more investigations are required in order to clarify their specificity and improve their efficiency by revealing their molecular targets and mechanism of action. Further clinical studies should confirm their ability to cure jaundice and standardize their medical inference.

## Conclusion

In this review we discussed in details some of the most important plants used for the treatment of jaundice. The remarkable potential of medicinal plants used in the context of gastrointestinal disorders is already well known and their utilization is quite common in ethnomedicine, however, the experimental verifications are limited. Some of the salient features are the beneficial effects in context of jaundice seen on the serum enzymes particularly in the AST, SGOT, SGPT and total bilirubin clearance. Growing number of clinical studies reveal the importance of particular plant species and their active compounds, which serve as basis for further drug development.

More studies are required in order to improve the efficiency and specificity of jaundice treatment based on ethnopharmacological knowledge and to standardize the clinical procedures. The broad spectrum of biologically active compounds could allow additional applications beyond jaundice cure, like gastrointestinal disorders, blood pressure and so on. The high efficiency and low number of side effects by using plant constituents indicates the ethnopharmacological approach of jaundice treatment as a high priority for future research.

## Author Contributions

DT, AM, EP, ZM, YL, and AA have written the first draft of the manuscript. AS, SN, LH, and JH revised and improved the first draft. All authors have seen and agreed on the finally submitted version of the manuscript.

## Conflict of Interest Statement

The authors declare that the research was conducted in the absence of any commercial or financial relationships that could be construed as a potential conflict of interest.

## Abbreviations

ACE: angiotensin converting enzyme
Ach: acetylcholine
ALP: alkaline phosphatase
ALT: alanine transaminase
AR: aldose reductase
AspAT: aspartate aminotransferase
AST: aspartate transaminase
ATP: adenosine triphosphate
AUC: area under the concentration time curve
cAMP: cyclic adenosine monophosphate
CaOX: calcium oxalate
CCL_4_: carbon tetrachloride
CED: *Caenorhabditis elegans* cell-death gene
CGT: ceramide galactosyltransferase
*C*_max_: maximum plasma concentration
COX: cyclooxygenase
CrMP: chromium mesoporphyrin
CYP: cytochromes P450
CYP2E1: Cytochrome P450 2E1
D-Gal N: D-galactosamine
DTD: DT-diaphorase
ERK: extracellular signal–regulated kinase
ET-NANB: enteric transmitted non-A non-B
GABA: gamma-Aminobutyric acid (γ-Aminobutyric acid)
GOT: glutamic oxaloacetic transaminase
GPT: glutamic–pyruvic transaminase
GSH: glutathione stimulation hormone
GST: glutathione *S*-transferase
HAV: hepatitis A virus
HBV: hepatitis B virus
HCV: hepatitis C virus
HDV: hepatitis D virus/hepatitis delta virus
HEV: hepatitis E virus
HIV: human immunodeficiency virus
HO 1: heme oxygenase 1
ICE: interleukin-1beta-converting enzyme
INH: isoniazid
JNK: c-Jun N-terminal kinases
LDH: lactate dehydrogenase
LOX: lipoxygenase
LPS: Lipopolysaccharide
LR: renal clearance
MAPK: mitogen-activated protein kinases
MDA: malondialdehyde
mRNA: messenger RNA
NA: noradrenaline
Na^+^ K^+^-ATPase: sodium potassium adenosine triphosphatase
NANBH non-A: non-B hepatitis
NK1: neurokinin 1 OTC over the counter PROD pentoxyresorufin *O*-dealkylase
PZA: pyrazinamide RBC red blood cells
RIF: rifampin
ROS: reactive oxygen species
RT: reverse transcriptase
SGOT: serum glutamic oxaloacetic transaminase
SnMP: tin mesoporphyrin
SOD: superoxide dismutase
*t*_1/2β_: elimination half life
*t*-BHP: *tert*-butyl hydroperoxide
TCM: Traditional Chinese medicine
TK: tyrosine kinase *t*_max_ maximum time
TNF-α: tumor necrosis factor- α
UDPGT: uridine diphosphoglucuronyl transferase
UGT: uridine 5′-diphospho-glucuronosyltransferase (UDP- glucuronosyltransferase)
wh. pl.: whole plant
WHO: World Health Organization
WW II: World War II.

## References

[B1] Agarwal K. Dhir H. Sharma A. Talukder G. (1992). The efficacy of two species of *Phyllanthus* in counteracting nickel clastogenicity. *Fitoterapia* 63 49–54.

[B2] Akbarsha M. A. Murugaian P. (2000). Aspects of the male reproductive toxicity/male antifertility property of andrographolide in albino rats: effect on the testis and the cauda epididymidal spermatozoa. *Phytother. Res.* 14 432–435. 10.1002/1099-1573(200009)14:6<432::AID-PTR622>3.0.CO;2-I 10960897

[B3] Alcaraz M. J. Jiménez M. J. (1988). Flavonoids as anti-inflammatory agents. *Fitoterapia* 59 25–38.

[B4] Al-Qarawi A. A. Abdel-Rahman H. A. El-Mougy S. A. (2001). Hepatoprotective activity of licorice in rat liver injury models. *J. Herbs Spices Med. Plants* 8 7–14. 10.1300/J044v08n01_02

[B5] Anand K. K. Singh B. Saxena A. K. Chandan B. K. Gupta V. N. (1994). Hepatoprotective studies of a fraction from the fruits of *Terminalia belerica* Roxb. On experimental liver injury in rodents. *Phytother. Res.* 8 287–292. 10.1002/ptr.2650080507

[B6] Angulo P. Patel T. Jorgensen R. A. Therneau T. M. Lindor K. D. (2000). Silymarin in the treatment of patients with primary biliary cirrhosis with a suboptimal response to ursodeoxycholic acid. *Hepatology* 32 897–900. 10.1053/jhep.2000.18663 11050036

[B7] Arase Y. Ikeda K. Murashima N. Chayama K. Tsubota A. Koida I. (1997). The long term efficacy of glycyrrhizin in chronic hepatitis C patients. *Cancer* 79 1494–1500. 10.1002/(SICI)1097-0142(19970415)79:8<1494::AID-CNCR8>3.0.CO;2-B 9118029

[B8] Arseculeratne S. N. Gunatilaka A. A. L. Panabokke R. G. (1985). Studies on medicinal plants of Sri Lanka. Part 14: toxicity of some traditional medicinal herbs. *J. Ethnopharmacol.* 13 323–335. 10.1016/0378-8741(85)90078-9 4058035

[B9] Asl M. N. Hosseinzadeh H. (2008). Review of pharmacological effects of Glycyrrhiza sp. and its bioactive compounds. *Phytother. Res.* 22 709–724. 10.1002/ptr.2362 18446848 PMC7167813

[B10] Bachmann T. L. Ghia F. Torssell K. B. G. (1993). Lignans and lactones from *Phyllanthus anisolobus*. *Phytochemistry* 33 189–191. 10.1016/0031-9422(93)85420-V

[B11] Bagalkotkar G. Sagineedu S. R. Saad M. S. Stanslas J. (2006). Phytochemicals from *Phyllanthus niruri* Linn. and their pharmacological properties: a review. *J. Pharm. Pharmacol.* 58 1559–1570. 10.1211/jpp.58.12.0001 17331318

[B12] Barzaghi N. Crema F. Gatti G. Pifferi G. Perucca E. (1990). Pharmacokinetic studies on IdB 1016, a silybin-phosphatidylcholine complex, in healthy human subjects. *Eur. J. Drug Metab. Pharmacokinet.* 15 333–338. 10.1007/BF03190223 2088770

[B13] Beretz A. Anton R. Stoclet J. C. (1978). Flavonoid compounds are potent inhibitors of cyclic AMP phosphodiesterase. *Experientia* 34 1054–1055. 10.1007/BF01915343 212288

[B14] Blumenthal M. Goldberg A. Brinkmann J. (2000). *Herbal Medicine, Expanded Commission E Monographs Cd-Rom.* Austin, TX: American Botanical Council.

[B15] Bunout D. Hirsch S. Petermann M. De La Maza M. P. Silva G. Kelly M. (1992). Controlled study of the effect of silymarin on alcoholic liver disease. *Rev. Med. Chil* 120 1370–1375. 1343377

[B16] Cai Y. Xu Y. Chan H. F. Fang X. He C. Chen M. (2016). Glycyrrhetinic acid mediated drug delivery carriers for hepatocellular carcinoma therapy. *Mol. Pharm.* 13 699–709. 10.1021/acs.molpharmaceut.5b00677 26808002

[B17] Calabrese C. Berman S. H. Babish J. G. Ma X. Shinto L. Dorr M. (2000). A phase I trial of andrographolide in HIV positive patients and normal volunteers. *Phytother. Res.* 14 333–338. 10.1002/1099-1573(200008)14:5<333::AID-PTR584>3.0.CO;2-D 10925397

[B18] Calixto J. B. Santos A. R. S. Filho V. C. Yunes R. A. (1998). A review of the plants of the genus *Phyllanthus*: and therapeutic potential. *Med. Res. Rev.* 18 225–258. 10.1002/(SICI)1098-1128(199807)18:4<225::AID-MED2>3.0.CO;2-X 9664291

[B19] Campos A. H. Schor N. (1999). *Phyllanthus niruri* inhibits calcium oxalate endocytosis by renal tubular cells: its role in urolithiasis. *Nephron* 81 393–397. 10.1159/000045322 10095174

[B20] Chander R. Srivastava V. Tandon And J. S. Kapoor N. K. (1995). Antihepatotoxic activity of diterpenes of *Andrographis paniculata* (Kal-Megh) against *Plasmodium berghei*-induced hepatic damage in *Mastomys natalensis*. *Int. J. Pharmacogn.* 33 135–138. 10.3109/13880209509055213

[B21] Chatterjee A. Pakrashi S. C. (1991). *The Treatise on Indian Medicinal Plants*, Vol. 1. New Delhi: Publications and Information Directorate, CSIR, 172.

[B22] Choi M.-K. Kim H.-G. Han J.-M. Lee J.-S. Lee J. S. Chung S. H. (2015). Hepatoprotective effect of *Terminalia chebula* against t-BHP-induced acute liver injury in C57/BL6 mice. *Evid. Based Complement. Alternat. Med.* 2015:517350 10.1155/2015/517350 PMC4321673 25691908

[B23] Chopra R. N. Nayar S. L. Chopra I. C. Asolkar L. V. Kakkar K. K. Chakre O. J. (1956). *Glossary of Indian medicinal plants; [with] Supplement.* New Delhi: Council of Scientific & Industrial Research.

[B24] Choudhury B. R. Poddar M. K. (1984). Andrographolide and kalmegh (*Andrographis paniculata*) extract: in vivo and in vitro effect on hepatic lipid peroxidation. *Methods Find. Exp. Clin. Pharmacol.* 6 481–485. 6513681

[B25] Chturvedi G. N. Tomar G. S. Tiwari S. K. Singh K. P. (1983). Clinical studies on Kalmegh (*Andrographis paniculata* nees) in infective hepatitis. *Anc. Sci. Life* 2 208–215. 22556984 PMC3336768

[B26] Cruz T. Galvez J. Ocete M. A. Crespo M. E. Sanchez de Medina L.-H. F. Zarzuelo A. (1998). Oral administration of rutoside can ameliorate inflammatory bowel disease in rats. *Life Sci.* 62 687–695. 10.1016/S0024-3205(97)01164-8 9472728

[B27] Cruz-Vega D. Verde-Star M. J. Salinas-Gonzalez N. R. Rosales-Hernandez B. Estrada-Garcia I. Mendez-Aragon P. (2009). Review of pharmacological effects of *Glycyrrhiza radix* and its bioactive compounds. *Zhongguo Zhong Yao Za Zhi.* 22 557–559. 20209894

[B28] Dafni A. Yaniv Z. Palevitch D. (1984). Ethnobotanical survey of medicinal plants in northern Israel. *J. Ethnopharmacol.* 10 295–310. 10.1016/0378-8741(84)90017-5 6748708

[B29] Dastagir G. Rizvi M. A. (2016). *Glycyrrhiza glabra* L.(Liquorice). *Pak. J. Pharm. Sci* 29 1727–1733. 27731836

[B30] Deng W. L. Nie R. J. Liu J. Y. (1982). Comparison of pharmacological effect of four andrographolides. *Yaoxue Tongbao* 17 195–198.

[B31] Dhar M. L. Dhar M. M. Dhawan B. N. Mehrotra B. N. Roy C. (1968). Screening of Indian plants for biological activity: Part 1. *Indian J. Exp. Biol.* 6 232–247. 5720682

[B32] Dhir H. Roy A. K. Sharma A. Talukder G. (1990). Protection afforded by aqueous extracts of Phyllanthus species against cytotoxicity induced by lead and aluminium salts. *Phytother. Res.* 4 172–176. 10.1002/ptr.2650040503

[B33] Dias M. A. Campos A. H. Cechinel Filho V. Yunes R. A. C. J. (1995). Analysis of the mechanisms underlying the contractile response induced by the hydroalcoholic extract of *Phyllanthus urinaria* in the guinea-pig urinary bladder in-vitro. *J. Pharm. Pharmacol.* 47 846–851. 10.1111/j.2042-7158.1995.tb05752.x 8583354

[B34] Dixit S. P. Achar M. P. Thabrew M. R. (1982). *Phyllanthus niruri* (Bhumyamalaki) and jaundice in children. *J. Natl. Integ. Med. Ass.* 25 269–272.

[B35] Duarte J. Perez-Vizcaino F. Zarzuelo A. Jimenez J. Tamargo J. (1993). Vasodilator effects of quercetin in isolated rat vascular smooth muscle. *Eur. J. Pharmacol.* 239 1–7. 10.1016/0014-2999(93)90968-N 8223884

[B36] Eerdunbayaer Orabi M. A. A. Aoyama H. Kuroda T. Hatano T. (2014). Structures of new phenolics isolated from licorice, and the effectiveness of licorice phenolics on vancomycin-resistant Enterococci. *Molecules* 19 13027–13041. 10.3390/molecules190913027 25157467 PMC6271213

[B37] Epstein M. T. Espiner E. A. Donald R. A. Hughes H. (1977). Effect of eating liquorice on the renin-angiotensin aldosterone axis in normal subjects. *Br. Med. J.* 1 488–490. 10.1136/bmj.1.6059.488 837172 PMC1605097

[B38] Evans W. C. (2009). *Trease and Evans’ Pharmacognosy.* Amsterdam: Elsevier.

[B39] Feher J. Deák G. Muzes G. (1989). Hepatoprotective activity of silymarin (Legalon) therapy in patients with chronic liver disease. *Orv. Hetil.* 130 2723–2727. 2574842

[B40] Ferenci P. Dragosics B. Dittrich H. Frank H. Benda L. Lochs H. (1989). Randomized controlled trial of silymarin treatment in patients with cirrhosis of the liver. *J. Hepatol.* 9 105–113. 10.1016/0168-8278(89)90083-4 2671116

[B41] Filho V. C. Santos A. R. S. Campos R. O. P. Miguel O. G. Yunes R. A. Ferrari F. (1996). Chemical and pharmacological studies of *Phyllanthus caroliniensis* in Mice. *J. Pharm. Pharmacol.* 48 1231–1236. 10.1111/j.2042-7158.1996.tb03928.x 9004183

[B42] Finney R. S. H. Somers G. F. (1958). The antiinflammatory activity of glycyrrhetinic acid and derivatives. *J. Pharm. Pharmacol.* 10 613–620. 10.1111/j.2042-7158.1958.tb10349.x 13588543

[B43] Fleming T. (1998). *PDR for Herbal Medicines.* Montvale, NJ: Medical Economics Company.

[B44] Flora K. Hahn M. Rosen H. Benner K. (1998). Milk thistle (*Silybum marianum*) for the therapy of liver disease. *Am. J. Gastroenterol.* 93 139–143. 10.1111/j.1572-0241.1998.00139.x 9468229

[B45] Flora K. D. Rosen H. R. Benner K. G. (1996). The use of naturopathic remedies for chronic liver disease. *Am. J. Gastroenterol.* 91 2654–2655. 8947022

[B46] Foo L. Y (1995). Amariinic acid and related ellagitannins from Phyllanthus amarus. *Phytochemistry* 39 217–224. 10.1016/0031-9422(94)00836-I

[B47] Foo L. Y. Wong H. (1992). Phyllanthusiin D, an unusual hydrolysable tannin from *Phyllanthus amarus*. *Phytochemistry* 31 711–713. 10.1016/0031-9422(92)90071-W

[B48] Foster S. (1991). *Milk thistle: Silybum marianum. Botanical Series, (305).* Austin, TX: American Botanical Council.

[B49] Fu B. Li S. Yu X. Yang P. Yu G. Feng R. (2010). Chinese ecosystem research network: progress and perspectives. *Ecol. Complex* 7 225–233. 10.1016/j.ecocom.2010.02.007

[B50] Fujita T. Sezik E. Tabata M. Yesilada E. Honda G. Takeda Y. (1995). Traditional medicine in Turkey VII. Folk medicine in middle and west Black Sea regions. *Econ. Bot.* 49 406–422. 10.1007/BF02863092

[B51] Fukai T. Tantai L. Nomura T. (1996). Isoprenoid-substituted flavonoids from *Glycyrrhiza glabra*. *Phytochemistry* 43 531–532. 10.1016/0031-9422(96)00307-X

[B52] Gálvez J. Cruz T. Crespo E. Ocete M. A. Lorente M. D. de Medina F. S. (1997). Rutoside as mucosal protective in acetic acid-induced rat colitis. *Planta Med.* 63 409–414. 10.1055/s-2006-957723 9342943

[B53] Gálvez J. Sánchez de Medina F. Jiménez J. Torres M. I. Fernández M. I. Núñez M. C. (1995). Effect of quercitrin on lactose-induced chronic diarrhoea in rats. *Planta Med.* 61 302–306. 10.1055/s-2006-958088 7480174

[B54] Gazak R. Walterova D. Kren V. (2007). Silybin and silymarin–new and emerging applications in medicine. *Curr. Med. Chem.* 14 315–338. 10.2174/092986707779941159 17305535

[B55] Girach R. D. Aminuddin Siddiqui P. A. Khan S. A. (1994). Traditional plant remedies among the Kondh of district Dhenkanal (Orissa). *Int. J. Pharmacogn.* 32 274–283. 10.3109/13880209409083005

[B56] Greive M. (1981). *A Modern Herbal*, Vol. 2 New York, NY: Dover Publications

[B57] Gulati R. K. Agarwal S. Agrawal S. S. (1995). Hepatoprotective studies on *Phyllanthus emblica* Linn. and quercetin. *Indian J. Exp. Biol.* 33 261–268. 7558182

[B58] Gupta S. Choudhry M. A. Yadava J. N. S. Srivastava V. Tandon J. S. (1990). Antidiarrhoeal activity of diterpenes of *Andrographis paniculata* (Kal-Megh) against *Escherichia coli* enterotoxin in in vivo models. *Int. J. Crude Drug Res.* 28 273–283. 10.3109/13880209009082833

[B59] Handa S. S. Sharma A. (1990). Hepatoprotective activity of andrographolide from *Andrographis paniculata* against carbontetrachloride. *Indian J. Med. Res.* 92 276–283. 2228074

[B60] Harnisch G. Stolze H. (1983). “Silybum marianum: mariendistel,” in *Bewaehrte Flanzendrogen in Wissenschaft Und Medizin* (Melsungen: Notamed Verlag), 203–215.

[B61] Harrison P. M. (1977). Ferritin: an iron-storage molecule. *Semin. Hematol.* 14 55–70. 318769

[B62] Hidaka I. Hino K. Korenaga M. Gondo T. Nishina S. Ando M. (2007). Stronger Neo-Minophagen CTM, a glycyrrhizin-containing preparation, protects liver against carbon tetrachloride-induced oxidative stress in transgenic mice expressing the hepatitis C virus polyprotein. *Liver Int.* 27 845–853. 10.1111/j.1478-3231.2007.01492.x 17617128

[B63] Hino K. Sainokami S. Shimoda K. Iino S. Wang Y. Okamoto H. (1994). Genotypes and titers of hepatitis C virus for predicting response to interferon in patients with chronic hepatitis C. *J. Med. Virol.* 42 299–305. 10.1002/jmv.1890420318 7516422

[B64] Hiramatsu N. Hayashi N. Katayama K. Mochizuki K. Kawanishi Y. Kasahara A. (1994). Immunohistochemical detection of Fas antigen in liver tissue of patients with chronic hepatitis C. *Hepatology* 19 1354–1359. 10.1002/hep.1840190606 7514559

[B65] Hobbs C. (1987). *Milk Thistle: The Liver Herb.* Capitola, CA: Botanica Press.

[B66] Huo H. Z. Wang B. Liang Y. K. Bao Y. Y. Gu Y. (2011). Hepatoprotective and antioxidant effects of licorice extract against CCl4-induced oxidative damage in rats. *Int. J. Mol. Sci.* 12 6529–6543. 10.3390/ijms12106529 22072903 PMC3210994

[B67] Hussain R. A. Dickey J. K. Rosser M. P. Matson J. A. Kozlowski M. R. Brittain R. J. (1995). A novel class of non-peptidic endothelin antagonists isolated from the medicinal herb *Phyllanthus niruri*. *J. Nat. Prod.* 58 1515–1520. 10.1021/np50124a006

[B68] Ikeda K. Arase Y. Kobayashi M. Saitoh S. Someya T. Hosaka T. (2006). A long-term glycyrrhizin injection therapy reduces hepatocellular carcinogenesis rate in patients with interferon-resistant active chronic hepatitis C: a cohort study of 1249 patients. *Dig. Dis. Sci.* 51 603–609. 10.1007/s10620-006-3177-0 16614974

[B69] Ikeda K. Saitoh S. Koida I. Arase Y. Tsubota A. Chayama K. (1993). A multivariate analysis of risk factors for hepatocellular carcinogenesis: a prospective observation of 795 patients with viral and alcoholic cirrhosis. *Hepatology* 18 47–53. 10.1002/hep.1840180109 7686879

[B70] Iruretagoyena M. I. Tobar J. A. González P. A. Sepúlveda S. E. Figueroa C. A. Burgos R. A. (2004). Andrographolide interferes with T cell activation and reduces experimental autoimmune encephalomyelitis in the mouse. *J. Pharm. Exp. Ther.* 312 366–372. 10.1124/jpet.104.072512 15331658

[B71] Ishikawa S. Saito T. (1980). The effect of glycyrrhetinic acid on the action of aldosterone in stimulating sodium transport in frog skin. *Endocrinol. Jpn.* 27 697–701. 10.1507/endocrj1954.27.697 6973465

[B72] Ishimaru K. Yoshimatsu K. Yamakawa T. Kamada H. Shimomura K. (1992). Phenolic constituents in tissue cultures of *Phyllanthus niruri*. *Phytochemistry* 31 2015–2018. 10.1016/0031-9422(92)80352-F

[B73] Jarukamjorn K. Don-in K. Makejaruskul C. Laha T. Daodee S. Pearaksa P. (2006). Impact of *Andrographis paniculata* crude extract on mouse hepatic cytochrome P450 enzymes. *J. Ethnopharmacol.* 105 464–467. 10.1016/j.jep.2005.11.024 16406417

[B74] Jeong H. G. You H. J. Park S. J. Moon A. R. Chung Y. C. Kang S. K. (2002). Hepatoprotective effects of 18β-glycyrrhetinic acid on carbon tetrachloride-induced liver injury: inhibition of cytochrome P450 2E1 expression. *Pharmacol. Res.* 46 221–227. 10.1016/S1043-6618(02)00121-4 12220964

[B75] Juang L. Sheu S. Lin T. (2004). Determination of hydrolyzable tannins in the fruit of *Terminalia chebula* Retz. by high-performance liquid chromatography and capillary electrophoresis. *J. Sep. Sci.* 27 718–724. 10.1002/jssc.200401741 15387468

[B76] Kapil A. Koul I. B. Banerjee S. K. Gupta B. D. (1993). Antihepatotoxic effects of major diterpenoid constituents of *Andrographis paniculata*. *Biochem. Pharmacol.* 46 182–185. 10.1016/0006-2952(93)90364-3 8347130

[B77] Ketterer B. (1988). Protective role of glutathione and glutathione transferases in mutagenesis and carcinogenesis. *Mutat. Res.* 202 343–361. 10.1016/0027-5107(88)90197-2 3057366

[B78] Khatoon S. Rai V. Rawat A. K. S. Mehrotra S. (2006). Comparative pharmacognostic studies of three Phyllanthus species. *J. Ethnopharmacol.* 104 79–86. 10.1016/j.jep.2005.08.048 16236476

[B79] Kimura M. Watanabe H. Abo T. (1992). Selective activation of extrathymic T cells in the liver by glycyrrhizin. *Biotherapy* 5 167–176. 10.1007/BF02171049 1419465

[B80] Kitagawa I. Chen W. Z. Hori K. Harada E. Yasuda N. Yoshikawa M. (1994). Chemical studies of Chinese licorice-roots. I. elucidation of five new flavonoid constituents from the roots of *Glycyrrhiza glabra* L. collected in Xinjiang. *Chem. Pharm. Bull.* 42 1056–1062. 10.1248/cpb.42.1056 8069956

[B81] Kokate C. K. Purohit A. P. Gokhale S. B. (2003). *Test Book of Pharmacognosy.* Pune: Nirali Prakashan.

[B82] Kumar R. A. Sridevi K. Kumar N. V. Nanduri S. Rajagopal S. (2004). Anticancer and immunostimulatory compounds from *Andrographis paniculata*. *J. Ethnopharmacol.* 92 291–295. 10.1016/j.jep.2004.03.004 15138014

[B83] Kusirisin W. Srichairatanakool S. Lerttrakarnnon P. Lailerd N. Suttajit M. Jaikang C. (2009). Antioxidative activity, polyphenolic content and anti-glycation effect of some Thai medicinal plants traditionally used in diabetic patients. *Med. Chem.* 5 139–147. 10.2174/157340609787582918 19275712

[B84] Lau G. K. Tsiang M. Hou J. Yuen S. Carman W. F. Zhang L. (2000). Combination therapy with lamivudine and famciclovir for chronic hepatitis B–infected Chinese patients: a viral dynamics study. *Hepatology* 32 394–399. 10.1053/jhep.2000.9143 10915748

[B85] Lee C. D. Ott M. Thyagarajan S. P. Shafritz D. A. Burk R. D. Gupta S. (1996). Phyllanthus amarus down-regulates hepatitis B virus mRNA transcription and replication. *Eur. J. Clin. Invest.* 26 1069–1076. 10.1046/j.1365-2362.1996.410595.x 9013081

[B86] Lee H.-S. Won N. H. Kim K. H. Lee H. Jun W. Lee K.-W. (2005). Antioxidant effects of aqueous extract of *Terminalia chebula* in vivo and in vitro. *Biol. Pharm. Bull.* 28 1639–1644. 10.1248/bpb.28.1639 16141531

[B87] Lee J. R. Park S. J. Lee H.-S. Jee S. Y. Seo J. Kwon Y. K. (2009). Hepatoprotective activity of licorice water extract against cadmium-induced toxicity in rats. *J. Evid. Based Complement. Altern. Med.* 6 195–201. 10.1093/ecam/nem078 PMC2686628 18955229

[B88] Lee S. Hyun P. Kim S. Kim K. Lee S. Kim B. (2005). Suppression of the onset and progression of collagen-induced arthritis by chebulagic acid screened from a natural product library. *Arthritis Rheum.* 52 345–353. 10.1002/art.20715 15641090

[B89] Leng-Peschlow E. (1994). Alcohol-related liver diseases-use of Legalon^®^. *Z. Klin. Med.* 2 22–27.

[B90] Li Z. Li Q. Jiang X. Zhang K. Guan R. (2014). Isolation and preparation of gallic acid from *Terminalia chebula* Retz. with high-speed counter-current chromatography. *Se Pu* 32 1404–1408. 25902651 10.3724/sp.j.1123.2014.07025

[B91] Lin C. N. Tome W. P. (1988). Antihepatotoxic principles of Sambucus formosana. *Planta Med.* 54 223–224. 10.1055/s-2006-962410 3174860

[B92] Lin G. Nnane I. P. Cheng T.-Y. (1999). The effects of pretreatment with glycyrrhizin and glycyrrhetinic acid on the retrorsine-induced hepatotoxicity in rats. *Toxicon* 37 1259–1270. 10.1016/S0041-0101(98)00263-3 10400287

[B93] Lindahl M. Tagesson C. (1993). Selective inhibition of group II phospholipase A2 by quercetin. *Inflammation* 17 573–582. 10.1007/BF00914195 8225564

[B94] Loguercio C. Festi D. (2011). Silybin and the liver: from basic research to clinical practice. *World J. Gastroenterol.* 17 2288–2301. 10.3748/wjg.v17.i18.2288 21633595 PMC3098397

[B95] Luper S. (1998). A review of plants used in the treatment of liver disease: part 1. *Altern. Med. Rev.* 3 410–421. 9855566

[B96] Mabberley D. J. (2008). *Mabberley’s Plant-book: A Portable Dictionary of Plants, Their Classification and Uses.* Cambridge: Cambridge University Press.

[B97] Magliulo E. Gagliardi B. Fiori G. P. (1978). Results of a double blind study on the effect of silymarin in the treatment of acute viral hepatitis, carried out at two medical centres (author’s transl). *Med. Klin.* 73 1060–1065. 353464

[B98] Mao X. Wu L.-F. Guo H.-L. Chen W.-J. Cui Y.-P. Qi Q. (2016). The genus Phyllanthus: an ethnopharmacological, phytochemical, and pharmacological review. *J. Evid. Based Complement. Altern. Med.* 2016:7584952. 10.1155/2016/7584952 PMC4854999 27200104

[B99] Meister A. (1994). Glutathione, ascorbate, and cellular protection. *Cancer Res.* 54(7 Suppl.), 1969s–1975s. 8137322

[B100] Meyer H. S. Eldredge J. D. Hogan R. (1999). Herbal medicines. The complete german commission e monographs: therapeutic guide to herbal medicines. *JAMA* 281 1852–1853. 10.1001/jama.281.19.1852-JBK0519-2-1

[B101] Mhaskar K. S. Blatter E. Caius J. F. (2000). *Kirtikar and Basu’s Illustrated Indian Medicinal Plants: Their Usage in Ayurveda and Unani Medicines.* New Delhi: Sri Satguru Publications.

[B102] Miadonna A. Tedeschi A. Leggieri E. Lorini M. Froldi M. Zanussi C. (1987). Effects of silybin on histamine release from human basophil leucocytes. *Br. J. Clin. Pharmacol.* 24 747–752. 10.1111/j.1365-2125.1987.tb03241.x 2449903 PMC1386399

[B103] Mita E. Hayashi N. Iio S. Takehara T. Hijioka T. Kasahara A. (1994). Role of fas ligand in apoptosis induced by hepatitis C virus infection. *Biochem. Biophys. Res. Commun.* 204 468–474. 10.1006/bbrc.1994.2483 7980502

[B104] Miyakawa Y. Iino S. (2001). Toward prevention of hepatocellular carcinoma developing in chronic hepatitis C. *J. Gastroenterol. Hepatol.* 16 711–714. 10.1046/j.1440-1746.2001.02543.x 11446875

[B105] Morales M. A. Lozoya X. (1994). Calcium-antagonist effects of quercetin on aortic smooth muscle. *Planta Med.* 60 313–317. 10.1055/s-2006-959491 7938264

[B106] Morazzoni P. Bombardelli E. (1995). *Silybum marianum* (*Carduus marianus*). *Fitoterapia* 66 3–42.

[B107] Morton J. F. (1981). *Atlas of Medicinal Plants of Middle America: Bahamas to Yucatan.* Springfield: Charles C. Thomas, 458–462.

[B108] Mourelle M. Muriel P. Favari L. Franco T. (1989). Prevention of CCl4-induced liver cirrhosis by silymarin. *Fundam. Clin. Pharmacol.* 3 183–191. 10.1111/j.1472-8206.1989.tb00449.x 2548940

[B109] Naik A. D. Juvekar A. R. (2003). Effects of alkaloidal extract of *Phyllanthus niruri* on HIV replication. *Indian J. Med. Sci.* 57 387. 14515028

[B110] Nakamura T. Fujii T. Ichihara A. (1985). Enzyme leakage due to change of membrane permeability of primary cultured rat hepatocytes treated with various hepatotoxins and its prevention by glycyrrhizin. *Cell Biol. Toxicol.* 1 285–295. 10.1007/BF00118193 3916986

[B111] Nanduri S. Nyavanandi V. K. Thunuguntla S. S. R. Kasu S. Pallerla M. K. Ram P. S. (2004). Synthesis and structure-activity relationships of andrographolide analogues as novel cytotoxic agents. *Bioorg. Med. Chem. Lett.* 14 4711–4717. 10.1016/j.bmcl.2004.06.090 15324893

[B112] Nomura T. Fukai T. Akiyama T. (2002). Chemistry of phenolic compounds of licorice (*Glycyrrhiza* species) and their estrogenic and cytotoxic activities. *Pure Appl. Chem.* 74 1199–1206. 10.1351/pac200274071199

[B113] Nose M. Ito M. Kamimura K. Shimizu M. Ogihara Y. (1994). A comparison of the antihepatotoxic activity between glycyrrhizin and glycyrrhetinic acid. *Planta Med.* 60 136–139. 10.1055/s-2006-959435 8202565

[B114] Ody P. (2000). *The Complete Guide Medicinal Herbal.* London: Dorling Kindersley.

[B115] Ogata T. Higuchi H. Mochida S. Matsumoto H. Kato A. Endo T. (1992). HIV-1 reverse transcriptase inhibitor from *Phyllanthus niruri*. *AIDS Res. Hum. Retroviruses* 8 1937–1944. 10.1089/aid.1992.8.1937 1283310

[B116] Ohno H. Miyoshi S. Araho D. Kanamoto T. Terakubo S. Nakashima H. (2014). Efficient utilization of licorice root by alkaline extraction. *In Vivo* 28 785–794. 25189890

[B117] Ohuchi K. Tsurufuji A. (1982). A study of the anti-inflammatory mechanism of glycyrrhizin. *Mino. Med. Rev.* 27 188–193.

[B118] Oliver-Bever B. (1983). Medicinal plants in tropical west africa III. Anti-infection therapy with higher plants. *J. Ethnopharmacol.* 9 1–83. 10.1016/0378-8741(83)90028-4 6668951

[B119] Owoyele B. V. Olaleye S. B. Oke J. M. Elegbe R. A. (2001). Anti-Inflammatory and analgesic activities of leaf extracts of *Landolphia owariensis*. *Afr. J. Biomed. Res.* 4 131–133.

[B120] Padmalatha K. Jayaram K. Raju N. L. Prasad M. N. V. Arora R. (2009). Ethnopharmacological and biotechnological significance of *Vitex*. *Biorem. Biodiv. Bioavail.* 3 6–14. 10.3329/bjsir.v43i3.1149

[B121] Panunto W. Jaijoy K. Lerdvuthisopon N. Lertprasertsuke N. Jiruntanat N. Soonthornchareonnon N. (2010). Acute and chronic toxicity studies of the water extract from dried fruits of Terminalia chebula Rezt. in rats. *Int. J. Appl. Res. Nat. Prod.* 3 36–43.

[B122] Parés A. Planas R. Torres M. Caballería J. Viver J. M. Acero D. (1998). Effects of silymarin in alcoholic patients with cirrhosis of the liver: results of a controlled, double-blind, randomized and multicenter trial. *J. Hepatol.* 28 615–621. 10.1016/S0168-8278(98)80285-7 9566830

[B123] Pathak D. Pathak K. Singla A. K. (1991). Flavonoids as medicinal agents-recent advances. *Fitoterapia* 62 371–389.

[B124] Perry L. M. Metzger J. (1980). *Medicinal Plants of East and Southeast Asia, Attributed Properties and Uses.* Cambridge, MA: MIT Press, 149–151.

[B125] Pettit G. R. Cragg G. M. Gust D. Brown P. Schmidt J. M. (1982a). The structures of phyllanthostatin 1 and phyllanthoside from the Central American tree *Phyllanthus acuminatus* Vahl. *Can. J. Chem.* 60 939–941.

[B126] Pettit G. R. Cragg G. M. Herald D. L. Schmidt J. M. Lohavanijaya P. (1982b). Isolation and structure of combretastatin. *Can. J. Chem.* 60 1374–1376. 10.1139/v82-202

[B127] Piva R. Penolazzi L. Borgatti M. Lampronti I. Lambertini E. Torreggiani E. (2009). Apoptosis of human primary osteoclasts treated with molecules targeting nuclear factor-κB. *Ann. N. Y. Acad. Sci.* 1171 448–456. 10.1111/j.1749-6632.2009.04906.x 19723088

[B128] Polya G. M. Wang B. H. Foo L. Y. (1995). Inhibition of signal-regulated protein kinases by plant-derived hydrolysable tannins. *Phytochemistry* 38 307–314. 10.1016/0031-9422(94)00547-7 7772301

[B129] Pradhan S. C. Girish C. (2006). Hepatoprotective herbal drug, silymarin from experimental pharmacology to clinical medicine. *Indian J. Med. Res.* 124 491–504. 17213517

[B130] Prakash A. Satyan K. S. Wahi S. P. Singh R. P. (1995). Comparative hepatoprotective activity of three *Phyllanthus* species, *P. urinaria*, *P. niruri* and *P. simplex*, on carbon tetrachloride induced liver injury in the rat. *Phytother. Res.* 9 594–596. 10.1002/ptr.2650090813

[B131] Pramyothin P. Udomuksorn W. Poungshompoo S. Chaichantipyuth C. (1994). Hepatoprotective effect of *Andrographis paniculata* and its constituent, andrographolide, on ethanol hepatotoxicity in rats. *Asia Pac. J. Pharmacol.* 9 73–78.

[B132] Puri A. Saxena R. Saxena R. P. Saxena K. C. Srivastava V. Tandon J. S. (1993). Immunostimulant agents from *Andrographis paniculata*. *J. Nat. Prod.* 56 995–999. 10.1021/np50097a002 8377022

[B133] Qian-Cutrone J. Huang S. Trimble J. Li H. Lin P. F. Alam M. (1996). Niruriside, a new HIV REV/RRE binding inhibitor from *Phyllanthus niruri*. *J. Nat. Prod.* 59 196–199. 10.1021/np9600560 8991954

[B134] Rajurkar N. S. Pardeshi B. M. (1997). Analysis of some herbal plants from India used in the control of diabetes mellitus by NAA and AAS techniques. *Appl. Radiat. Isot.* 48 1059–1062. 10.1016/S0969-8043(97)00103-6 9394437

[B135] Ramellini G. Meldolesi J. (1974). Stabilization of isolated rat liver plasma membranes by treatment in vitro with silymarin. *Arzneimittelforschung* 24 806–808. 4408361

[B136] Raphael T. J. Kuttan G. (2003). Effect of naturally occurring triterpenoids glycyrrhizic acid, ursolic acid, oleanolic acid and nomilin on the immune system. *Phytomedicine* 10 483–489. 10.1078/094471103322331421 13678231

[B137] Row L. R. Srinivasulu C. Smith M. Rao G. S. R. S. (1966). Crystalline constituents of euphorbiaceae-V. *Tetrahedron* 22 2899–2908. 10.1016/S0040-4020(01)99083-0

[B138] Rush G. F. Gorski J. R. Ripple M. G. Sowinski J. Bugelski P. Hewitt W. R. (1985). Organic hydroperoxide-induced lipid peroxidation and cell death in isolated hepatocytes. *Toxicol. Appl. Pharmacol.* 78 473–483. 10.1016/0041-008X(85)90255-8 4049396

[B139] Sabouri Ghannad M. Mohammadi A. Safiallahy S. Faradmal J. Azizi M. Ahmadvand Z. (2014). The effect of aqueous extract of *Glycyrrhiza glabra* on herpes simplex virus 1. *Jundishapur J. Microbiol.* 7:e11616 10.5812/jjm.11616 PMC4216581 25368801

[B140] Salmi H. Sarna S. (1982). Effect of silymarin on chemical, functional, and morphological alterations of the liver: a double-blind controlled study. *Scand. J. Gastroenterol.* 17 517–521. 10.3109/00365528209182242 6753109

[B141] Sanchez de Medina F. Vera B. Galvez J. Zarzuelo A. (2002). Effect of quercitrin on the early stages of hapten induced colonic inflammation in the rat. *Life Sci.* 70 3097–3108. 10.1016/S0024-3205(02)01568-0 12008093

[B142] Santos A. R. Filho V. C. Niero R. Viana A. M. Moreno F. N. Campos M. M. (1994). Analgesic effects of callus culture extracts from selected species of *Phyllanthus* in mice. *J. Pharm. Pharmacol.* 46 755–759. 10.1111/j.2042-7158.1994.tb03897.x 7837046

[B143] Saraswat B. Visen P. K. S. Patnaik G. K. Dhawan B. N. (1995). Effect of andrographolide against galactosamine-induced hepatotoxicity. *Fitoterapia* 66 415–420.

[B144] Sarkar R. Hazra B. Mandal N. (2012). Reducing power and iron chelating property of *Terminalia chebula* (Retz.) alleviates iron induced liver toxicity in mice. *BMC Complement. Altern. Med.* 12:144 10.1186/1472-6882-12-144 PMC3489879 22938047

[B145] Satomi Y. Nishino H. Shibata S. (2005). Glycyrrhetinic acid and related compounds induce G1 arrest and apoptosis in human hepatocellular carcinoma HepG2. *Anticancer Res.* 25 4043–4047. 16309197

[B146] Schuppan D. Jia J. Brinkhaus B. Hahn E. G. (1999). Herbal products for liver diseases: a therapeutic challenge for the new millennium. *Hepatology* 30 1099–1104. 10.1002/hep.510300437 10498665

[B147] Sharma A. Rathore H. S. (2010). Prevention of acetaminophen induced hepatorenal toxicity in mice with fruits of *Terminalia chebula* (Myrobalan). *Thai. J. Toxicol.* 25 144–153.

[B148] Sharma A. Singh R. T. Sehgal V. Handa S. S. (1991). Antihepatotoxic activity of some plants used in herbal formulations. *Fitoterapia* 62 131–138. 10.1016/j.fitote.2015.01.001

[B149] Shebl R. I. Amin M. A. Emad-Eldin A. Bin Dajem S. M. Mostafa A. S. Ibrahim E. H. (2012). Antiviral activity of liquorice powder extract against varicella zoster virus isolated from Egyptian patients. *Chang Gung Med. J.* 35 231–239. 22735054 10.4103/2319-4170.106149

[B150] Shen Y.-C. Chen C.-F. Chiou W.-F. (2002). Andrographolide prevents oxygen radical production by human neutrophils: possible mechanism(s) involved in its anti-inflammatory effect. *Br. J. Pharmacol.* 135 399–406. 10.1038/sj.bjp.0704493 11815375 PMC1573154

[B151] Shibata S. (2000). A drug over the millennia: pharmacognosy, chemistry, and pharmacology of licorice. *J. Pharm. Soc. Jpn.* 120 849–862. 10.1248/yakushi1947.120.10_849 11082698

[B152] Shiki Y. Shirai K. Saito Y. Yoshida S. H. O. Mori Y. Wakashin M. (1992). Effect of glycyrrhizin on lysis of hepatocyte membranes induced by anti-liver cell membrane antibody. *J. Gastroenterol. Hepatol.* 7 12–16. 10.1111/j.1440-1746.1992.tb00927.x 1543863

[B153] Shimizu M. Horie S. Terashima S. Ueno H. Hayashi T. Arisawa M. (1989). Studies on aldose reductase inhibitors from natural products. II. Active components of a Paraguayan crude drug “Para-parai mi,” *Phyllanthus niruri*. *Chem. Pharm. Bull.* 37 2531–2532. 10.1248/cpb.37.2531 2514047

[B154] Shisheva A. Shechter Y. (1992). Quercetin selectively inhibits insulin receptor function in vitro and the bioresponses of insulin and insulinomimetic agents in rat adipocytes. *Biochemistry* 31 8059–8063. 1324726 10.1021/bi00149a041

[B155] Šimanek V. Kren V. Ulrichová J. Vicar J. Cvak L. (2000). Silymarin: what is in the name? An appeal for a change of editorial policy. *Hepatology* 32 442–444. 10.1021/bi00149a041 10960282

[B156] Singh R. P. Banerjee S. Rao A. R. (2001). Modulatory influence of *Andrographis paniculata* on mouse hepatic and extrahepatic carcinogen metabolizing enzymes and antioxidant status. *Phytother. Res.* 15 382–390. 10.1053/jhep.2000.9770 11507728

[B157] Srividya N. Periwal S. (1995). Diuretic, hypotensive and hypoglycaemic effect of *Phyllanthus amarus*. *Indian J. Exp. Biol.* 33 861–864. 10.1002/ptr.730 8786163

[B158] Suolinna E. M. Lang D. R. Racker E. (1974). Quercetin, an artificial regulator of the high aerobic glycolysis of tumor cells. *J. Natl. Cancer Inst.* 53 1515–1519. 4279302 10.1093/jnci/53.5.1515

[B159] Suzuki H. (1983). Effects of glycyrrhizin on biochemical tests in patients with chronic hepatitis. *Double Blind Trial Asian Med. J.* 26 423–438. 10.1093/jnci/53.5.1515

[B160] Suzuki H. Ohta Y. Takino T. Fujisawa K. Hirayama C. Shimizu N. (1977). The therapeutic effects of stronger neo minophagen C for chronic hepatitis. *Igaku No Ayumi* 102 562–568.

[B161] Syamasundar K. V. Singh B. Thakur R. S. Husain A. Kiso Y. Hikino H. (1985). Antihepatotoxic principles of *Phyllanthus niruri* herbs. *J. Ethnopharmacol.* 14 41–44. 4087921 10.1016/0378-8741(85)90026-1

[B162] Takahara T. Watanabe A. Shiraki K. (1994). Effects of glycyrrhizin on hepatitis B surface antigen: a biochemical and morphological study. *J. Hepatol.* 21 601–609. 10.1016/S0168-8278(94)80108-8 7814808

[B163] Tanaka N. Yamamura Y. Santa T. Kotaki H. Uchino K. Sawada Y. (1993). Pharmacokinetic profiles of glycyrrhizin in patients with chronic hepatitis. *Biopharm. Drug Dispos.* 14 609–614. 10.1016/S0168-8278(94)80108-8 8251615

[B164] Tănăsescu C. Petrea S. Băldescu R. Macarie E. Chiriloiu C. Purice S. (1987). Use of the Romanian product Silimarina in the treatment of chronic liver diseases. *Med. Interne* 26 311–322. 10.1002/bdd.2510140707 3072661

[B165] Tandon A. Tandon B. N. Bhujwala R. A. (2001). Treatment of subacute hepatitis with lamivudine and intravenous glycyrrhizin: a pilot study. *Hepatol. Res.* 20 1–8. 10.1016/S1386-6346(00)00123-6 11282481

[B166] Tandon A. Tandon B. N. Bhujwala R. A. (2002). Clinical spectrum of acute sporadic hepatitis E and possible benefit of glycyrrhizin therapy. *Hepatol. Res.* 23 55–61. 10.1016/S1386-6346(01)00155-3 12084556

[B167] Tasaduq S. A. Singh K. Sethi S. Sharma S. C. Bedi K. L. Singh J. (2003). Hepatocurative and antioxidant profile of HP-1, a polyherbal phytomedicine. *Hum. Exp. Toxicol.* 22 639–645. 10.1191/0960327103ht406oa 14992325

[B168] Tasduq S. A. Singh K. Satti N. K. Gupta D. K. Suri K. A. Johri R. K. (2006). *Terminalia chebula* (fruit) prevents liver toxicity caused by sub-chronic administration of rifampicin, isoniazid and pyrazinamide in combination. *Hum. Exp. Toxicol.* 25 111–118. 10.1191/0960327106ht601oa 16634329

[B169] Thai Pharmacopoeia Committee (1995). Thai herbal pharmacopoeia. *Depart. Med. Sci.* 1 51–56.

[B170] Thyagarajan S. P. Thirunalasundari T. Subramanian S. Venkateswaran P. S. Blumberg B. S. (1988). Effect of phyllanthus amarus on chronic carriers of hepatitis b virus. *Lancet* 332 764–766. 10.1016/S0140-6736(88)92416-6 2901611

[B171] Trivedi N. Rawal U. M. (2000). Hepatoprotective and toxicological evaluation of *Andrographis paniculata* on severe liver damage. *Ind. J. Pharmacol.* 32 288–293.

[B172] Uesato S. Kitagawa Y. Kamishimoto M. Kumagai A. Hori H. Nagasawa H. (2001). Inhibition of green tea catechins against the growth of cancerous human colon and hepatic epithelial cells. *Cancer Lett.* 170 41–44. 10.1016/S0304-3835(01)00571-7 11448533

[B173] Ullah M. Khan M. U. Mahmood A. Malik R. N. Hussain M. Wazir S. M. (2013). An ethnobotanical survey of indigenous medicinal plants in Wana district south Waziristan agency. *Pak. J. Ethnopharmacol.* 150 918–924. 10.1016/j.jep.2013.09.032 24120747

[B174] Ulmann A. Menard J. Corvol P. (1975). Binding of glycyrrhetinic acid to kidney mineralocorticoid and glucocorticoid receptors. *Endocrinology* 97 46–51. 10.1210/endo-97-1-46 166832

[B175] Umarani D. Devaki T. Govindaraju P. Shanmugasundaram K. R. (1985). Ethanol induced metabolic alterations and the effect of *Phyllanthus niruri* in their reversal. *Anc. Sci. Life* 4 174–180. 22557474 PMC3331514

[B176] Unander D. W. Blumberg B. S. (1991). In vitro activity of *Phyllanthus* (Euphorbiaceae) species against the DNA polymerase of hepatitis viruses: effects of growing environment and inter- and intra-specific differences. *Econ. Bot.* 45 225–242. 10.1007/BF02862050

[B177] Unander D. W. Webster G. L. Blumberg B. S. (1990). Records of usage or assays in *Phyllanthus* (Euphorbiaceae) I. Subgenera Isocladus, Kirganelia, Cicca and Emblica. *J. Ethnopharmacol.* 30 233–264. 10.1016/0378-8741(90)90105-3 2259214

[B178] Unander D. W. Webster G. L. Blumberg B. S. (1991). Uses and bioassays in *Phyllanthus* (Euphorbiaceae): a compilation. II. The subgenus *Phyllanthus*. *J. Ethnopharmacol.* 34 97–133. 10.1016/0378-8741(91)90029-D 1795536

[B179] Unander D. W. Webster G. L. Blumberg B. S. (1992). Usage and bioassays in *Phyllanthus* (Euphorbiaceae): a compilation III. The subgenera *Eriococcus*, *Conami*, *Gomphidium*, *Botryanthus*, *Xylophylla* and *Phyllanthodendron*, and a complete list of the species cited in the three-part series. *J. Ethnopharmacol.* 36 103–112. 10.1016/0378-8741(92)90009-G 1608266

[B180] Unander D. W. Webster G. L. Blumberg B. S. (1995). Usage and bioassays in *Phyllanthus* (Euphorbiaceae). IV. Clustering of antiviral uses and other effects. *J. Ethnopharmacol.* 45 1–18. 10.1016/0378-8741(94)01189-7 7739222

[B181] Utsumi K. (1984). Action of glycyrrhizin on biomembrane [in Japanese]. *Proc. Symp. Liver Glycyrrhizin* 104.

[B182] van Rossum T. G. Vulto A. G. Hop W. C. Brouwer J. T. Niesters H. G. Schalm S. W. (1999). Intravenous glycyrrhizin for the treatment of chronic hepatitis C: a double-blind, randomized, placebo-controlled phase I/II trial. *J. Gastroenterol. Hepatol.* 14 1093–1099. 10.1046/j.1440-1746.1999.02008.x 10574137

[B183] van Rossum T. G. Vulto A. G. Hop W. C. Schalm S. W. (2001). Glycyrrhizin-induced reduction of ALT in European patients with chronic hepatitis C. *Am. J. Gastroenterol.* 96 2432–2437. 10.1111/j.1572-0241.2001.04049.x 11513186

[B184] Varshney I. P. Jain D. C. Srivastava H. C. (1983). Study of saponins from *Glycyrrhiza glabra* root. *Int. J. Crude Drug Res.* 21 169–172. 10.3109/13880208309070637

[B185] Veldt B. J. Hansen B. E. Ikeda K. Verhey E. Suzuki H. Schalm S. W. (2006). Long-term clinical outcome and effect of glycyrrhizin in 1093 chronic hepatitis C patients with non-response or relapse to interferon. *Scand. J. Gastroenterol.* 41 1087–1094. 10.1080/00365520600641365 16938723

[B186] Venkateswaran P. S. Millman I. Blumberg B. S. (1987). Effects of an extract from *Phyllanthus niruri* on hepatitis B and woodchuck hepatitis viruses: in vitro and in vivo studies. *Proc. Natl. Acad. Sci. U.S.A.* 84 274–278. 10.1073/pnas.84.1.274 3467354 PMC304186

[B187] Visen P. K. S. Shukia B. Patnaik G. K. Dhawan B. N. (1993). Andrographolide protects rat hepatocytes against paracetamol-induced damage. *J. Ethnopharmacol.* 40 131–136. 10.1016/0378-8741(93)90058-D 8133653

[B188] Wagner H. Diesel P. Seitz M. (1974). The chemistry and analysis of silymarin from *Silybum marianum* Gaertn. *Arzneimittelforschung* 24 466–471. 4408125

[B189] Wakamatsu T. Nakahashi Y. Hachimine D. Seki T. Okazaki K. (2007). The combination of glycyrrhizin and lamivudine can reverse the cisplatin resistance in hepatocellular carcinoma cells through inhibition of multidrug resistance-associated proteins. *Int. J. Oncol.* 31 1465–1472. 10.3892/ijo.31.6.1465 17982673

[B190] Wang B. E. (2000). Treatment of chronic liver diseases with traditional Chinese medicine. *J. Gastroenterol. Hepatol.* 15(Suppl.), E67–E70. 10.1046/j.1440-1746.2000.02100.x 10921385

[B191] Wang L. Yang R. Yuan B. Liu Y. Liu C. (2015). The antiviral and antimicrobial activities of licorice, a widely-used Chinese herb. *Acta Pharm. Sin. B* 5 310–315. 10.1016/j.apsb.2015.05.005 26579460 PMC4629407

[B192] Wang M. Cheng H. Li Y. Meng L. Zhao G. Mai K. (1995). Herbs of the genus *Phyllanthus* in the treatment of chronic hepatitis B: observations with three preparations from different geographic sites. *J. Lab. Clin. Med.* 126 350–352. 7561442

[B193] Wang Y. Kong L. Chen Y. (2005). Behavioural and biochemical effects of fractions prepared from Banxia houpu decoction in depression models in mice. *Phytother. Res.* 19 526–529. 10.1002/ptr.1697 16114088

[B194] Wang Z. Okamoto M. Kurosaki Y. Nakayama T. Kimura T. (1996). Pharmacokinetics of glycyrrhizin in rats with D-galactosamine-induced hepatic disease. *Biol. Pharm. Bull.* 19 901–904. 10.1248/bpb.19.901 8799498

[B195] Watari N. (1973). An electronmicroscopic study of the effects of glycyrrhizin on experimentally injured liver. *Mini Med. Rev.* 11 12–17.

[B196] Wellington K. Jarvis B. (2001). Silymarin: a review of its clinical properties in the management of hepatic disorders. *BioDrugs* 15 465–489. 10.2165/00063030-200115070-00005 11520257

[B197] WHO (1999). *WHO Monographs on Selected Medicinal Plants*, Vol. 2 Geneva: World Health Organization.

[B198] Williamson E. M. (2003). “Liquorice,” in *Potter’s Cyclopedia of Herbal Medicines*. ed. Daniels C. W. (Saffron Walden: C. W. Daniels), 269–271.

[B199] Wong W. Wu P. Yuen M. Cheng C. Yew W. Wong P. (2000). Antituberculosis drug-related liver dysfunction in chronic hepatitis B infection. *Hepatology* 31 201–206. 10.1002/hep.510310129 10613746

[B200] Xu H. (1986). *Oriental materia medica: a concise guide.* New Canaan, CT: Keats Publishing.

[B201] Yamamoto S. Maekawa Y. Imamura M. Hisajima T. (1958). Treatment of hepatitis with the antiallergic drug, Stronger Neo-Minophagen C. *Clin. Med. Pediatr.* 13:73.

[B202] Yamamura Y. Tanaka N. Santa T. Kotaki H. Aikawa T. Uchino K. (1995). The relationship between pharmacokinetic behaviour of glycyrrhizin and hepatic function in patients with acute hepatitis and liver cirrhosis. *Biopharm. Drug Dispos.* 16 13–21. 10.1002/bdd.2510160103 7711280

[B203] Yang R. Yuan B.-C. Ma Y.-S. Zhou S. Liu Y. (2016). The anti-inflammatory activity of licorice, a widely used Chinese Herb. *Pharm. Biol.* 55 5–18. 10.1080/13880209.2016.1225775 27650551 PMC7012004

[B204] Yarnell E. (1997). Botanical medicine for cystitis. *J. Altern. Complement. Med.* 3 269–275. 10.1089/act.1997.3.269

[B205] Yeasmin T. Akhter Q. S. Tasnim M. Jahan S. (2016). Hepatoprotective effect of *Terminalia chebula* (Haritaki) on serum bilirubin in paracetamol induced liver damage in wister albino rats. *Dinajpur. Med. Col. J.* 9 78–83.

[B206] Yeh S. F. Hong C. Y. Huang Y. L. Liu T. Y. Choo K. B. Chou C. K. (1993). Effect of an extract from *Phyllanthus amarus* on hepatitis B surface antigen gene expression in human hepatoma cells. *Antiviral Res.* 20 185–192. 10.1016/0166-3542(93)90019-F 8470882

[B207] Yoshikawa M. Matsui Y. Kawamoto H. Umemoto N. Oku K. Koizumi M. (1997). Effects of glycyrrhizin on immune-mediated cytotoxicity. *J. Gastroenterol. Hepatol.* 12 243–248. 10.1111/j.1440-1746.1997.tb00416.x 9142643

[B208] Yu J.-Y. Ha J. Y. Kim K.-M. Jung Y.-S. Jung J.-C. Oh S. (2015). Anti-Inflammatory activities of licorice extract and its active compounds, glycyrrhizic acid, liquiritin and liquiritigenin, in BV2 cells and mice liver. *Molecules* 20 13041–13054. 10.3390/molecules200713041 26205049 PMC6332102

