# Corrigendum: Ethnopharmacological Approaches for Therapy of Jaundice: Part II. Highly Used Plant Species from Acanthaceae, Euphorbiaceae, Asteraceae, Combretaceae, and Fabaceae Families

**DOI:** 10.3389/fphar.2017.00690

**Published:** 2017-10-06

**Authors:** Devesh Tewari, Andrei Mocan, Emil D. Parvanov, Archana N. Sah, Seyed M. Nabavi, Lukasz Huminiecki, Zheng Feei Ma, Yeong Yeh Lee, Jarosław O. Horbańczuk, Atanas G. Atanasov

**Affiliations:** ^1^Department of Pharmaceutical Sciences, Faculty of Technology, Kumaun University, Nainital, India; ^2^Department of Pharmaceutical Botany, “Iuliu Hatieganu” University of Medicine and Pharmacy, Cluj-Napoca, Romania; ^3^ICHAT and Institute for Life Sciences, University of Agricultural Sciences and Veterinary Medicine, Cluj-Napoca, Romania; ^4^Division BIOCEV, Institute of Molecular Genetics, Academy of Sciences of the Czech Republic, Prague, Czechia; ^5^Applied Biotechnology Research Center, Baqiyatallah University of Medical Sciences, Tehran, Iran; ^6^Institute of Genetics and Animal Breeding of the Polish Academy of Sciences, Jastrzebiec, Poland; ^7^School of Medical Sciences, Universiti Sains Malaysia, Kota Bharu, Malaysia; ^8^Department of Public Health, Xi'an Jiaotong-Liverpool University, Suzhou, China; ^9^Department of Pharmacognosy, University of Vienna, Vienna, Austria; ^10^Department of Vascular Biology and Thrombosis Research, Centre for Physiology and Pharmacology, Medical University of Vienna, Vienna, Austria

**Keywords:** jaundice, bilirubin, oxidative stress, traditional use, phytoconstituents, serum enzymes, alkaline phosphatase

In the original article, there was a mistake in the legend for Figure 4 as published (the spelling of isosilibin was incorrect). The correct legend appears below.

In the original article, there was a mistake in Figure 4 as published (CH_3_ group was missing in the Silybin structure). The corrected Figure 4 appears below.

**Figure 4:**
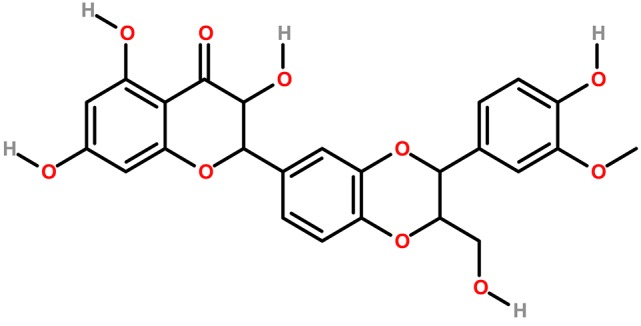
The chemical structures of phytoconstituents of Milk thistle **(A)** Silybin and **(B)** Isosilybin.

The authors apologize for these errors and state that this does not change the scientific conclusions of the article in any way.

## Conflict of interest statement

The authors declare that the research was conducted in the absence of any commercial or financial relationships that could be construed as a potential conflict of interest. The handling Editor declared a past co-authorship with the author SN.

